# Site-selective arylations of nature-inspired flavonoids or steroidal phenols *via* C—H or O—H activation

**DOI:** 10.1080/14756366.2025.2530615

**Published:** 2025-07-18

**Authors:** Rebeka Ignácz, Noémi Bózsity, Dénes Unger, Zoltán Kele, István Zupkó, Attila Hunyadi, Marija Gjorgoska, Tea Lanišnik Rižner, Erzsébet Mernyák

**Affiliations:** a Institute of Pharmacognosy, University of Szeged, Szeged, Hungary; b Institute of Pharmacodynamics and Biopharmacy, University of Szeged, Szeged, Hungary; c Department of Medicinal Chemistry, University of Szeged, Szeged, Hungary; d HUN-REN-SZTE Biologically Active Natural Products Research Group, Szeged, Hungary; e Institute of Biochemistry and Molecular Genetics, Faculty of Medicine, University of Ljubljana, Ljubljana, Slovenia

**Keywords:** 13α-oestrone, protoflavone, arylation, diaryliodonium salt, anticancer

## Abstract

Phenols are important structural elements of natural products and pharmaceuticals. Due to their versatile chemical transformability, phenols are frequently used building blocks in medicinal chemistry. Their aromatic nature allows directed C(sp^2^)—H functionalisations, especially at the *ortho* positions. In contrast, *meta* substitutions are less well known. As a continuation of our recently described metal-catalysed cross couplings, here we report arylations of two nature-inspired phenol derivatives *via* C—H or O—H activation. A directing group (DG) was introduced onto C-3-*O* of 13α-oestrone, and the resulting carbamate was subjected to Cu(II)-catalysed *meta* arylation using diaryliodonium triflates as reagents. As a result, C-1-arylated derivatives were obtained. The arylation of the 1’-*O*-butyl protoapigenone proceeded regioselectively at C-5-*O*. The 1-(4-*tert*-butylphenyl)-13α-oestrone carbamate and all *O*-arylated protoflavones substantially inhibited the growth of the applied human cancer cell lines and exerted proapoptotic activity on HeLa cells. The 1-(4-*tert*-butylphenyl)-13α-oestrone proved to be a potent 17β-HSD1 inhibitor.

## Introduction

**Figure 1.**
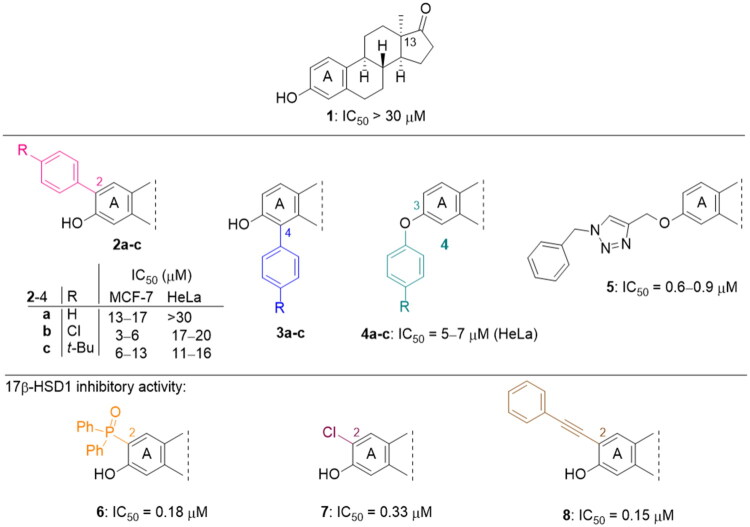
Structure of 13α-oestrone **1** and its recently identified potent cytotoxic arylated derivatives **1**–**5** ^6–8^ and selected 17β-HSD1 enzyme inhibitors **6**–**8** ^10^^,^^11^.

Phenol derivatives of natural origin possess remarkable biological activities^1^, and those with anticancer effect are particularly important. Oestrogens and flavonoids are characteristic examples. However, anti-tumour activities of their semi-synthetic derivatives often surpass those of their natural counterparts^2^^,^^3^. It is worth mentioning that a higher efficacy is usually associated with the extension of side effects. Consequently, increasing selectivity is one of the major challenges of anticancer drug developments. The one-step epimerisation of natural oestrone results in its hormonally inactive 13-epimer **1**, due to the conformational change in the steroid core (Figure 1)^4^^,^^5^. This semi-synthetic compound provides a promising scaffold for the development of oestrone-based anti-tumour lead molecules acting in a selective manner. According to previous studies, certain transformations at the phenolic ring of 13α-oestrone substantially influence its biological activity. Concerning cytotoxic action, arylations at C-2, C-4 or C-3-*O* are advantageous (Figure 1)^6–8^. The presence of the 4-chlorophenyl group at C-2 in compound **2** promotes the cell growth-inhibitory potential, especially against cancer cell lines HeLa and MCF-7 (cervical and breast carcinoma, respectively)^8^. A comparison of the results obtained for derivatives of the two regioisomers **2** and **3** suggests that the latter group is less active^6^. C-3-*O*-Arylated derivatives **4** proved to be selective against the HeLa cell line^7^. Additionally, we found that introduction of the *N*-benzyltriazolylmethyl moiety onto C-3-*O* of 13α-oestrone substantially improves the cytotoxic activity, resulting in submicromolar IC_50_ values for compound **5** ^9^.

We earlier investigated the inhibitory effects of certain A-ring substituted 13α-oestrone derivatives on some enzymes involved in oestradiol biosynthesis. 2-, 4- or 2,4-*bis*-Substituted aryl halides, aralkynyl, aralkyl or phosphonate derivatives were the subjects of investigation^6^^,^^10^^,^^11^. We found that the nature and the position of the substituent introduced markedly influences the inhibitory activity against the 17β-hydroxysteroid dehydrogenase 1 (17β-HSD1) enzyme, which catalyses the stereospecific reduction of oestrone to 17β-oestradiol. The 1.2 micromolar IC_50_ value (against 17β-HSD1) obtained for the basic 13α-oestrone compound **1** could be improved by one order of magnitude *via* certain *ortho* substitutions. It can be stated that derivatives substituted at position C-2 exerted more pronounced inhibitory activity with a few exceptions. A further important structure-activity relationship is that the presence of a free phenolic hydroxy group was found to be beneficial for most of the compounds. Assuming that the investigated biological effects of 13α-oestrone derivatives greatly depend on the substitution pattern of the A-ring, transformations at the *meta*-position would also be worth investigating.

**Figure 2.**
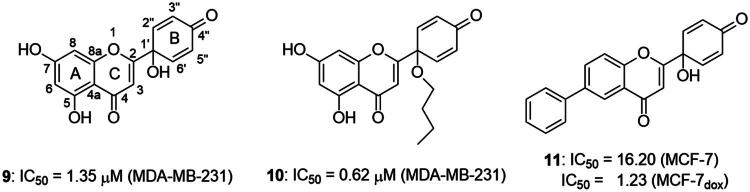
Structures of natural and semi-synthetic cytotoxic protoflavone derivatives **10**–**11**, and some characteristic IC_50_ values as reported previously^14–16^.

It is both particularly challenging and an urgent need to develop therapeutic approaches against triple-negative breast cancer (TNBC) ^12^. Regrettably, none of the previously reported A-ring arylated 13α-oestrone derivatives **1**–**5** displayed significant anticancer potential against the TNBC cell line MDA-MB-231. TNBC is commonly characterised by the absence of three receptor proteins: oestrogen and progesterone receptors (ER and PR, respectively) and the HER2 growth factor^13^. TNBC has a more aggressive course than other breast carcinomas and it generally develops at a younger age. TNBC subtypes require different treatments and their prognoses are poor. The chemotherapy followed by many side effects markedly reduces the quality of life of patients. Recently, novel approaches, namely utilisation of polyadenosine diphosphate-(ADP)-ribose polymerase inhibitors (PARPi) and immunotherapy, have come to the focus of attention,^12^. However, the use of PARP inhibitors still does not avoid side effects, and immunotherapy can only be used, when one of its target proteins is expressed on tumour cells. The above points suggest that further comprehensive research is needed to develop more effective and selective therapeutic preferences for TNBC. Protoapigenone **9** is a rare natural flavonoid that displays prominent anticancer effect with low toxicity *in vitro* and *in vivo*, and it is particularly active against MDA-MB-231 cells (Figure 2) ^14^^,^^15^. Its B-ring has been identified as a pharmacophore, since the presence of the dienone system is essential for its anti-tumour activity^15^. In addition, its semi-synthetic 1′-*O*-butyl derivative **10** is even more active (Figure 2)^15^. Concerning the outstanding submicromolar cytotoxic effect of compound **10**, it could provide a promising basis for the development of more potent drug candidates against TNBC.

Dankó et al. previously reported the synthesis of 6-phenyl protoflavone derivative **11** bearing no phenolic OH functions (Figure 2)^16^. Arylation at C-6 was achieved *via* Suzuki coupling of the bromoaryl compound with phenylboronic acid as a reagent. 6-Phenylated protoflavone **11** should be highlighted, because of its high selectivity towards MCF-7_Dox_ (MCF-7 breast carcinoma adapted to doxorubicin) versus non-resistant MCF-7. Such compounds can also bypass resistance caused by ABCB1 or ABCG2 efflux transporters, and thus overcome MDR (multidrug-resistant) cancer. Based on the above, introduction of the phenyl group onto C-6 has proved to be advantageous in several respects. However, the question arises, whether the directed arylation of dihydroxy derivative **10** is beneficial in terms of extent and/or selectivity of the anticancer effect.

C(sp^2^)–H bond functionalisation has undergone a major breakthrough over the last two decades^17^^,^^18^. Concerning ­phenol substrates, *ortho*-directed substitutions have received remarkable attention^19^^,^^20^. However, selective *meta*-functionalisation of the C(sp^2^)—H bond is still hardly known^21^. The electron-donating nature of the hydroxy group allows the modification of phenols at their *ortho*/*para* position, but it limits their *meta*-substitution. A significant improvement has been achieved in this field by elaboration of the palladium-catalysed “end-on template” ^22^ and the traceless directing group relay^23^ strategies. Nevertheless, there is a continuous search for new, more efficient and selective methodologies.

Diaryliodonium salts have recently emerged as inexpensive electrophilic arylation reagents, due to their efficient one-pot synthetic availability^24^. They have found extensive utilisation both in metal-catalysed and metal-free reactions. Maraswami et al. described a copper-catalysed *meta*-selective C(sp^2^)–arylation protocol for phenols, with high functional group tolerance^25^. They reacted phenyl carbamates (bearing the carbamoyl group as a DG) with diaryliodonium salts under Cu(II) catalysis to provide a *meta*-arylated product. The methodology elaborated for small-molecular substituted phenols was extended to the C-1-phenylation of oestrone carbamate. This was the first example in the literature for the *meta*-selective C—H arylation of oestrone derivatives. To the best of our knowledge, there are no literature reports for the *meta*-arylated 13α-oestrone compounds. Ciana et al. performed the phenylation of oestrone under similar reaction conditions^26^. Since the starting compound possessed a free phenolic hydroxy group, the reaction occurred at C-2 in a regioselective manner without affecting the *meta*-position.

*O*-Arylations of phenol derivatives to form diaryl ethers might also be achieved *via* Cu(II) catalysis^27^. These couplings require mild reaction conditions, and they can even be performed at room temperature. We recently described the direct arylation of 13α-oestrone at C-3-*O via* Chan–Lam coupling using arylboronic acids as reagents^7^. However, *O*-arylations can also be implemented in metal-free conditions^28^. Oxygen nucleophiles readily react with diaryliodonium salts under basic conditions. According to the proposed mechanism, the counteranion is a leaving group, while a nucleophilic attack occurs at the iodine centre of the neutral diaryliodonium salt.

The aforementioned arylation strategies suggest that phenolic compounds might react with diaryliodonium salts *via* different routes resulting in *ortho*-, *meta*-, *para*- or *O*-arylated products. The outcome of the reactions depends on the presence and the nature of the DG, on the substitution pattern of the aromatic ring and on the reaction conditions. Furthermore, the extension of these arylation procedures from small-molecular phenols to more complex natural derivatives might result in unexpected products.

With these considerations in mind, here we aimed to investigate the outcome of the arylation of 13α-oestrone and protoflavone derivatives **1** and **10** with diaryliodonium salt reagents under Cu(II) catalysis. We intended to explore chemo- and regioselectivities of the arylations starting from substrates with free phenolic hydroxy groups or with DGs introduced. The determination of the cytotoxic activity of the newly synthesised steroids and protoflavones was planned against human adherent cancer cell lines (HeLa, SiHa, C33A, MCF-7 and MDA-MB-231). The mechanistical study of the most potent antiproliferative derivative on the most sensitive cell line was designed, including cell cycle progression and mitochondrial membrane potential assay. We planned to determine the potential inhibitory effects of the newly synthesised steroidal compounds against the 17β-HSD1 enzyme.

## Materials and methods

Chemical syntheses, characterisation data of the reported compounds, as well as experimental conditions of the pharmacological assays performed are described in Supporting Information.

## Results and discussion

### Chemistry

Two nature-inspired steroid **1** and protoflavone **10** derivatives were chosen as substrates for arylation reactions, taking into account chemical and biological aspects. On the one hand, the two compounds might behave as *O*-nucleophiles, leading to *O*-arylated products. Note that the reactivity of their OH groups differs significantly. On the other hand, their C—H activation ability is greatly influenced by the position and the number of the OH groups on the aromatic rings. Protoflavone **10** has no free *meta*-position, hence it can only be substituted at its C—OH groups or at the *ortho* positions. However, 13α-oestrone **1** provides an opportunity to arylation at its C-3-*O* or at each aromatic C—H.

**Scheme 1.**
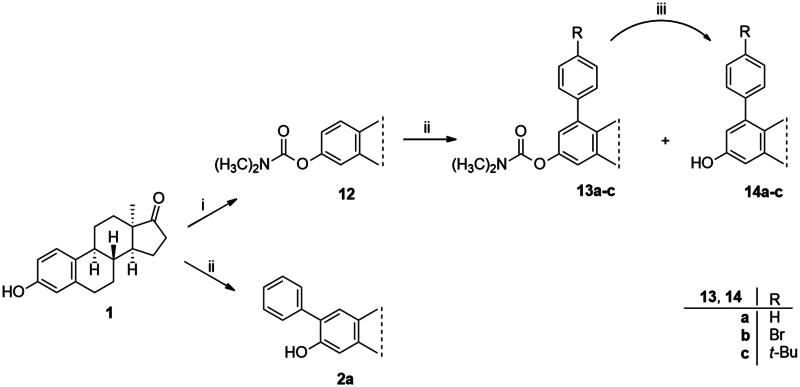
Synthesis of arylated 13α-oestrone derivatives **2a**, **13a**-**c** and **14a**-**c**. Reagents and conditions: (i) *N*,*N*-dimethylcarbamoyl chloride (1.0 equiv.), Cs_2_CO_3_ (2.0 equiv.), toluene, 100 °C, 2 h; (ii) iodonium salt (1.2 equiv.), copper(II) triflate (10 mol %), DCE, 70 °C, 3 h; (iii) sodium hydroxide (6 equiv.), isopropyl alcohol, 80 °C, 2 h.

The first type of transformation included the introduction of a carbamoyl DG onto the C-3-*O* of 13α-oestrone **1** as described earlier^29^. The carbamate derivative **12** was then subjected to *meta*-arylation with 1.2 equiv. of diphenyliodonium triflate reagent and 10 mol% Cu(II) triflate as a catalyst in 1,2-dichloroethane at 70 °C (Scheme 1). The reaction progress was monitored by thin layer chromatography (TLC). After the starting material has completely been transformed (3 h), the reaction mixture was taken up, and the crude product was subjected to purification by column chromatography. First, the entire mixture of 1-phenyl carbamate **13a** and its 3-OH derivative **14a** was isolated in order to determine their proportion. The ^1^H NMR investigation revealed that the product contained the desired compounds **13a** and **14a** in a 3:1 ratio. Thereafter the two arylated derivatives **13a** and **14a** were completely separable by column chromatography using gradient elution (hexanes/*tert*-butyl methyl ether (MTBE) from 9:1 (v/v) to 7:3 (v/v)).

Arylation of **12** with diphenyliodonium triflate reagent was also performed under the same conditions without using any catalyst. The un-catalysed reaction proceeded much slower and only traces of the products were detectable by TLC even after 24 h.

After the phenylation of the carbamate derivative **12**, reactions were extended to the synthesis of *meta*-arylated products with *para*-substituted C-1-aryl rings. Two diaryliodonium salts have been chosen: one bearing a bromine at the *para* position, and the other with an electron-donating, relatively large alkyl group. We described earlier that the presence of a halogen on the A-ring of the 13α-oestrone derivative might be beneficial concerning its biological activity^6^^,^^8^. In addition, bromoaryl derivatives allow subsequent modifications *via* transition metal-catalysed cross couplings. The introduction of the 4-*tert*-butylphenyl group is supported by the structure-activity relationship established previously. Namely compound **2c** exhibits a more pronounced cytotoxic activity in comparison with its 2-phenyl counterpart **2a**^6^. The *para*-substituted diaryliodonium salts reacted with chemo- and regioselectivity similar to the unsubstituted ones. The nature and the size of the substituent at C-4′ did not influence the reactions. The desired products **13b**,**c**; **14b**,**c** were obtained in high yields.

In order to produce the by-products **14a**–**c** in larger quantities, we elaborated an efficient method for hydrolysis of carbamates **13a**–**c**. The base-catalysed hydrolysis described in the literature^25^ was performed in ethanol. However, the solvent applied might be converted to its aldehyde counterpart under basic conditions, and it might react in a condensation reaction at C-16 of the steroid reaction partner. To avoid this side-reaction, we carried out the hydrolysis in isopropyl alcohol, using 6 equiv. of sodium hydroxide. The hydrolysis resulted in the corresponding 3-OH derivatives **14a**–**c** in high yields.

In the second type of modification, our aim was to investigate the chemo- and regioselectivity of steroid arylation without pre-functionalisation (no DG was introduced). In the test reaction, 13α-oestrone **1** was subjected to phenylation with 1.2 equiv. of the diphenyliodonium triflate reagent and 10 mol% Cu(II) triflate (3 h, at 70 °C in 1,2-dichloroethane). In this case, phenylation occurred exclusively at the C-2 *ortho* position, resulting in compound **2a**. This high regioselectivity is probably due to the larger steric hindrance at C-4 caused by the B ring. The product (**2a**) formed is identical to that obtained earlier by Suzuki coupling^6^. No transformation was observed in the reaction without catalyst.

**Scheme 2.**
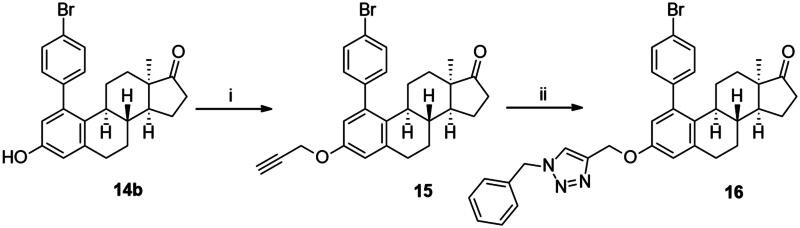
Introduction of the *N*-benzyltriazolylmethyl moiety onto the phenolic OH function. Reagents and conditions: (i) propargyl bromide (2 equiv. (80 wt% in toluene), K_2_CO_3_ (7 equiv.), 70 °C, 5 h; (ii) CuI (0.05 equiv.), Ph_3_P (0.1 equiv.), DIPEA (3 equiv.), toluene, reflux, 2 h.

In order to exploit the previously established structure–activity relationship^9^ in the present research, the 4-bromophenyl product **14b** was chosen for directed post-functionalisation (Scheme 2). The reason for this choice was that compound **14b** allows modifications not only at its phenolic OH function, but on the newly introduced aromatic ring, too. The presence of the bromoaryl moiety provides an opportunity to perform transition metal-catalysed cross coupling reactions at its C-4 position. Here we introduced the *N*-benzyltriazolylmethyl moiety onto C-3-*O*. The propargylation of 3-OH furnished a terminal alkyne **15** which was subjected to copper-catalysed azide–alkyne cycloaddition reaction (CuAAC) with benzyl azide as coupling partner. The desired triazol derivative **16** was obtained in high yield.

The structures of the newly synthesised arylated steroidal compounds **13**–**16** were evaluated from their ^1^H and ^13^C NMR spectra, using general knowledge of chemical shift dispersion and the aid of the ^1^H–^1^H coupling pattern.

**Scheme 3.**
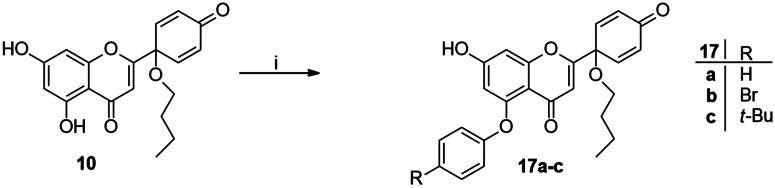
Arylation of protoflavone **10** *via* O–H activation. Reagents and conditions: (i) iodonium salt (1.2 equiv.), copper(II) triflate (10 mol %), DCE, 70 °C, 3 h.

In continuation of our work, protoflavone **10** was subjected to arylation with 1.2 equiv. diphenyliodonium triflate reagent and 10 mol% Cu(II) triflate as a catalyst (3 h, at 70 °C in 1,2-dichloroethane, Scheme 3). The substitution pattern of the aromatic ring allows here two reaction directions. The substrate **10** might behave as a C-nucleophile, reacting with the electrophilic arylation reagent at C-6 or C-8. Furthermore, acting as *O*-nucleophile, it can form diaryl ethers at its two hydroxy groups. Surprisingly, no C(sp^2^)-arylation occurred but *O*-phenylation did take place solely at 5-OH.

**Scheme 4.**
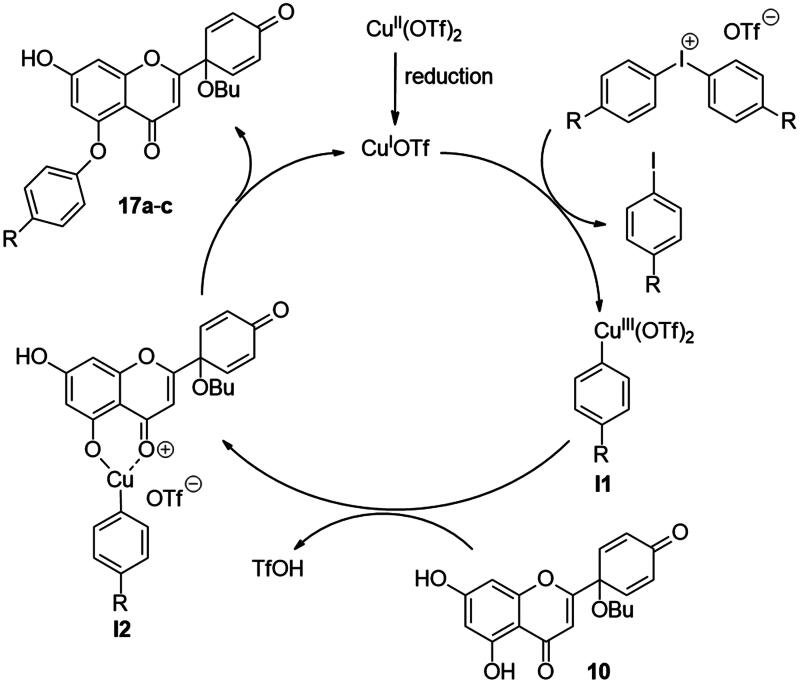
Proposed mechanism of the site-selective *O*-arylation of protoflavone **10**

The high regioselectivity observed for the etherification is an unexpected outcome, since the hydrogen bond formed between 5-OH and the neighbouring C = O generally limits the transformations of this hydroxy group. Nevertheless, the explanation for the etherification taking place on the less reactive hydroxy group is probably due to the characteristic complexation ability of the flavonoid^30^^,^^31^. Literature reveals formation of stable Cu(II) complexes of certain natural flavonoids, including apigenin. One of the Cu(II) binding sites is the structural moiety represented by the 5-OH and the C-4 carbonyl groups. The stability of such complexes has been confirmed by UV-Vis spectroscopy in agreement with TD-DFT calculation results^30^^,^^31^. Considering the structural similarities between apigenin and protoflavone **10**, complexation at the particular binding site in A- and C-rings might here also occur, which influences the reactivity of the phenolic hydroxy groups. Based on the proposed mechanism discussed in the literature^32^^,^^33^, it is plausible that Cu(II) is reduced to Cu(I) in the first step, followed by the oxidative addition of the diaryliodonium salt to the Cu(I) triflate in the next step of the catalytic cycle (Scheme 4). This results in intermediate **I1**, which forms chelate complex **I2** with protoflavone **10** by ligand exchange. The Cu(III) ion creates two bonds with the hydroxyketone moiety and third with the aryl group in **I2**. Transfer of the aryl group to C-5-*O* leads to the site-selective formation of a diaryl ether. The reaction was carried out without using a catalyst too, but no transformation was observed. To the best of our knowledge, there are no literature reports on regioselective *O*-activations at 5-OH of protoflavones *via* Cu(II)-catalysis.

The structures of the newly synthesised 5-*O*-arylated protoflavones **17a**–**c** were confirmed from ^1^H,^13^C NMR, COSY, HSQC and HMBC spectra. The characteristic 5-OH and 7-OH proton signals of flavonoids in DMSO-d_6_ solvent appear in range of 12–13 and 10–11 ppm, respectively^34^^,^^35^. The higher chemical shift belongs to the 5-OH group forming a hydrogen bond with the oxo function at C-4. In the ^1^H NMR spectra of compounds **17a**–**c** only a single OH signal was detected, at around 10.9 ppm. This indicates that the substitution occurred at 5-OH, and not at 7-OH. Our findings are consistent with those reported in the literature^35^.

In addition to NMR spectroscopic structural elucidation, UV-Vis spectroscopic analysis was also carried out. The UV-Vis spectrum of apigenin in methanol has two characteristic absorption bands. Band I with a maximum at 335 nm, and band II with a maximum at 266 nm^30^. The hydroxyketone moiety (5-OH and C-4 carbonyl) of apigenin forms a stabile complex with Al(III), which results in a batochromic shift of band II (about 30 nm)^36^. Here we recorded the UV-Vis spectra of protoflavone **10** and one of the arylated derivative **17b** alone and in the presence of Al(III) ion (Supporting Information Figure 1 A–D). The two absorption bands in spectrum of protoflavone **10** are displayed at maximums of around 230 and 300 nm. The complexation led to a batochromic shift of nearly 30 nm, which corresponds to that described in the literature for apigenin. The spectrum of arylated compound **17b** alone is similar to that of protoflavone **10** with two absorption bands. Nevertheless, mixing **17b** with AlCl_3_ solution did not cause a batochromic shift of band II. This provides further evidence that the hydroxy group on C-5 is not present in free form in compound **17b**.

### Pharmacology

We evaluated the cytotoxic activity of the newly synthesised arylated compounds **13**–**17** by the MTT assay^37^ on a panel of human adherent cancer cell lines. The panel included breast (MCF-7 and MDA MB-231) and cervical (HeLa, SiHa and C33A) cancer cell lines differing in receptorial or HPV status^38–41^. Tumour selectivity was determined on the NIH/3T3 mouse fibroblast cell line.

The newly synthesised steroid derivatives **13a**–**c**, **14a**–**c**, **16** displayed moderate growth-inhibitory potential against the investigated cancer cell lines, except for the *tert*-butylphenyl derivatives (Table 1). The 1–(4-*tert*-butylphenyl) carbamate **13c** exerted potent, low micromolar (IC_50_ = 4.74–10.10 µM) cytotoxic action against all investigated cell lines. Nevertheless, the IC_50_ values are slightly higher on receptor- or HPV-negative cells, compared to the ER-expressed breast and HPV-infected cervical cancer cells, respectively. The 3-OH derivative **14c** displays the same trend, but with significantly higher IC_50_ values (IC_50_ = 10.57–20.48 µM). The cytotoxic action of the 1–(4-bromophenyl) carbamate **13b** falls within the same range as that of compound **14c**, but with higher selectivity. In comparison with the data obtained for the *ortho*-arylated products described earlier^6^, *meta*-substitution has not produced better results generally. Even the hopeful introduction of the *N*-benzyltriazolylmethyl moiety on C-3-*O* (compound **16**) did not increase the antiproliferative effect. Despite all these findings, it should be mentioned, that the presence of the aryl group at C-1 can greatly influence the interaction of the compounds with key enzymes involved in steroid biosynthesis. As C-1-substituted oestrone derivatives are rarely found in the literature, this is an unexploited area from both the chemical and biological points of view. Consequently, inhibitory tests were performed against 17β-HSD1 enzyme (Table 2). The six *meta*-arylated steroid derivatives bearing carbamoyloxy **13a**–**c** or hydroxy moieties **14a**–**c** and the two previously synthesised *ortho* phenyl compounds **2-Ph** and **4-Ph** (Table 2) were subjected to investigations in two concentrations. Compounds bearing phenolic OH function **14a**–**c** exerted more potent inhibition, than their carbamate counterparts **13a**–**c**. These data correlate with those published earlier. ^10^^,^^11^ The 1-(*tert*-butylphenyl)-3-hydroxy derivative **14c** was found to be the most effective in the 10 µM test concentration with an inhibitory effect over 50%. Effects of the phenolic compounds **14a**–**c** did not show a significant difference at the higher concentration with their activity around 70%. Among all the *meta*-arylated derivatives, **14c** proved to be the most potent with an IC_50_ value of 2.7 µM (95% CI: 1.5–4.4). Our results show that introduction of a *tert*-butylphenyl moiety onto C-1 is preferable to that of the phenyl group. The earlier synthesised 2- or 4-phenylated compounds^6^ were also included in the studies to investigate the influence of the A-ring arylation pattern on 17β-HSD1 inhibition. The results suggest that there is no marked difference between the inhibition of 1- or 2-phenyl derivatives (in favour of the former), but introduction of phenyl group onto C-4 is less advantageous. The presence of a large group next to the B-ring results in a sterically hindered derivative, which presumably does not fit well into the appropriate binding site of the enzyme.

**Table 1.** Cytotoxic effects of the investigated steroid **13a**–**c**, **14a**–**c** and **16** and protoflavon compounds **17a**–**c**, measured by MTT assay after 72 h.

	Structure		Inhibition (%) (calculated IC_50_)^a^					
		[µM]	MCF-7	MDA-MB-231	HeLa	SiHa	C33A	NIH/3T3
**13a**	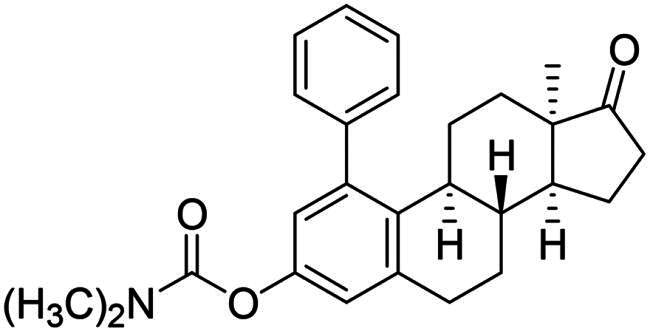	10	22.46	<10^b^	30.90	13.67	<10	13.47
		30	52.84	40.95	59.92	41.05	27.20	22.53
		IC_50_						
**14a**	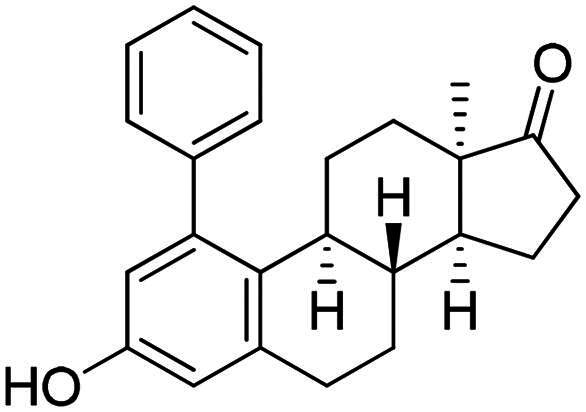	10	13.06	<10	16.12	<10	<10	13.47
		30	15.42	<10	43.01	<10	12.72	22.53
		IC_50_						
**13b**	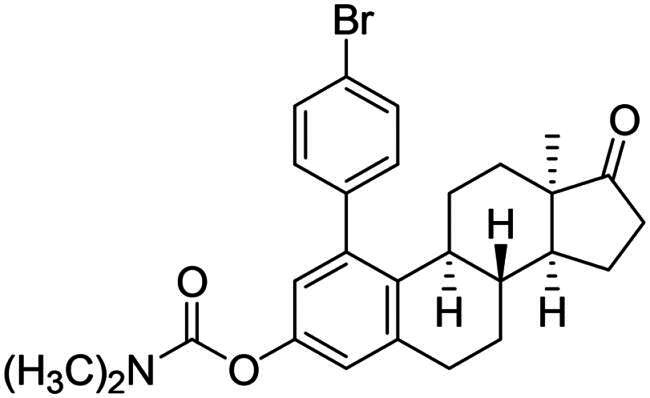	10	37.51	<10	53.57	38.62	<10	<10
		30	68.70	13.88	59.30	40.04	57.8	46.27
		IC_50_						
**14b**	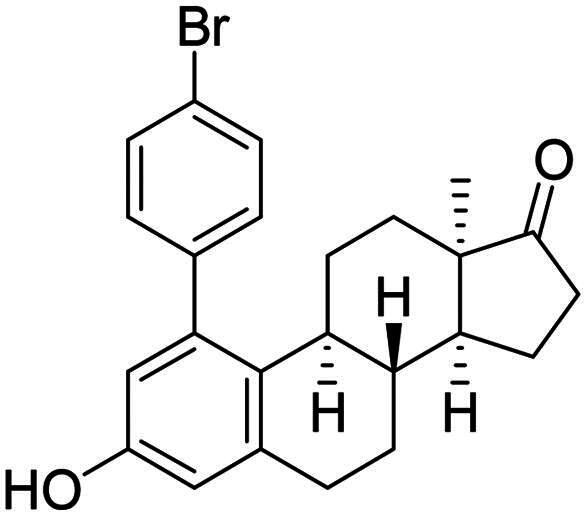	10	35.14	<10	26.53	<10	28.69	16.14
		30	81.51	46.84	71.56	59.67	56.95	41.70
		IC_50_	13.53		17.51			
**13c**	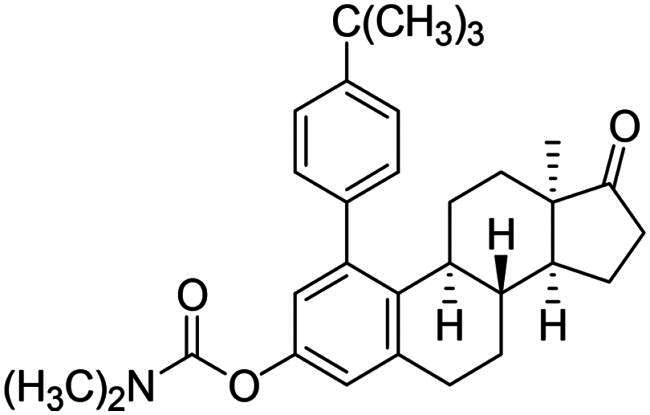	10	58.40	43.83	53.75	54.77	41.13	47.27
		30	101.63	92.86	98.64	100.96	93.59	97.67
		IC_50_	5.70	9.09	7.90	4.74	10.10	8.75
**14c**	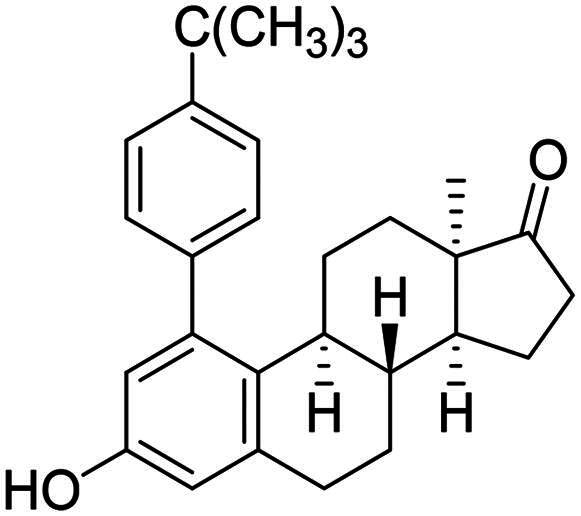	10	35.35	<10	21.92	20.16	25.41	16.12
		30	100.93	91.32	98.33	99.61	89.79	98.35
		IC_50_	10.57	20.48	13.02	12.49	14.42	13.74
**16**	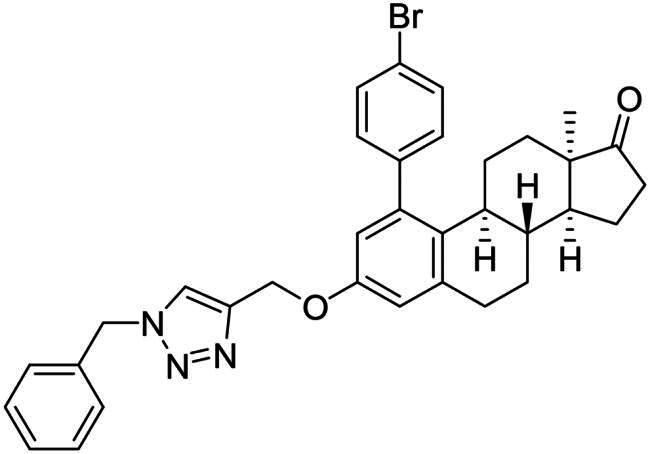	10	<10	<10	22.36	<10	<10	<10
		30	<10	<10	33.12	10.45	<10	11.15
		IC_50_						
**17a**	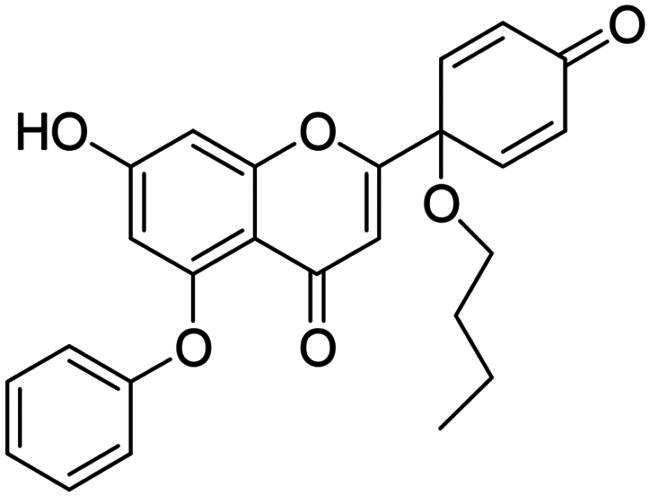	10	93.09	95.69	93.57	86.23	98.12	94.25
		30	93.73	96.86	94.27	94.95	98.55	98.67
		IC_50_	0.97	0.72	1.16	1.29	0.43	2.33
**17b**	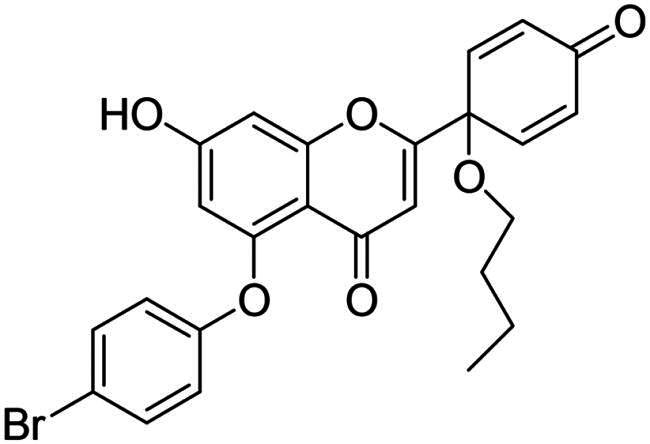	10	97.36	97.03	97.02	84.48	97.68	97.55
		30	98.39	97.87	97.70	93.82	97.80	98.57
		IC_50_	1.15	0.73	1.09	1.39	0.52	1.61
**17c**	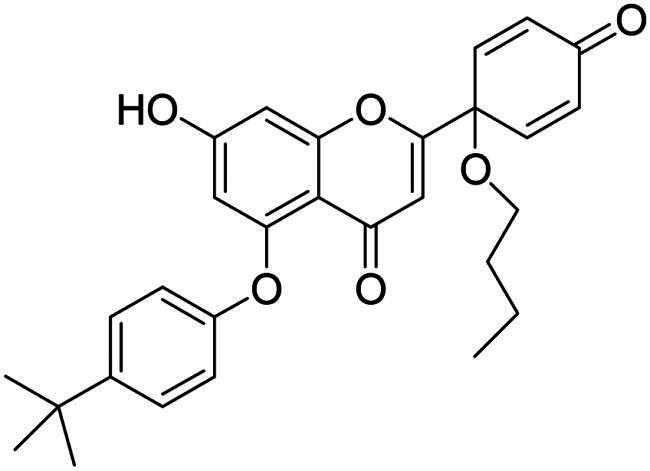	10	98.47	98.27	97.95	89.77	97.94	92.72
		30	98.94	96.96	98.03	96.24	97.90	98.99
		IC_50_	0.60	0.42	0.47	0.64	0.39	0.93
**10**	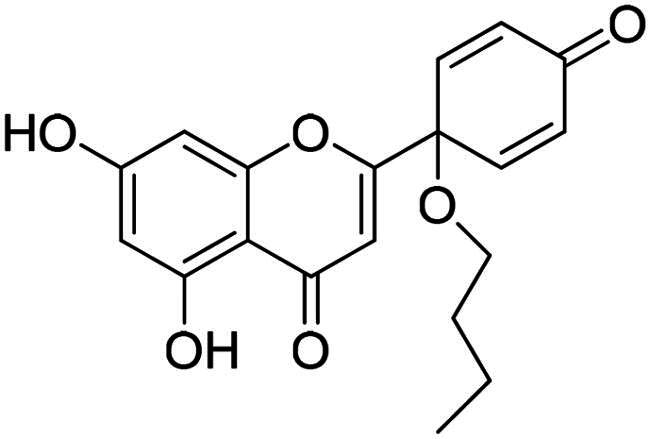	10	87.72	91.68	95.73	92.20	98.05	95.51
		30	93.34	92.79	96.23	96.34	98.49	99.06
		IC_50_	3.03	1.06	1.43	1.6	0.97	2.27
Cisplatin		IC_50_	5.8	19.1	12.4	7.8	3.7	4.7

Growth inhibitions are given in % after 10 and 30 µM treatment, and calculated IC_50_ values of the test compounds were determined, when the inhibition exceeded 70%.

^a^Mean value from two independent measurements with five parallel wells; relative standard deviation <20%.

^b^ Inhibition values <10% are not presented.

**Table 2.** 17β-HSD1 inhibitory effects of the investigated steroid derivatives (**13a**–**c** and **14a**–**c**).

	Structure	[µM]	Inhibition (% ± SD) (calculated IC_50_ (95% CI))^a^
**13a**	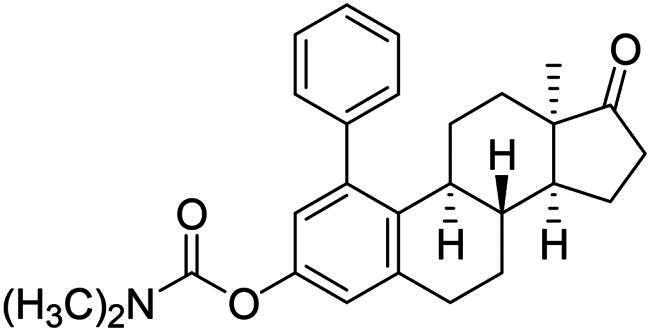	10	No inhibition
		100	33.6 ± 14.9
**14a**	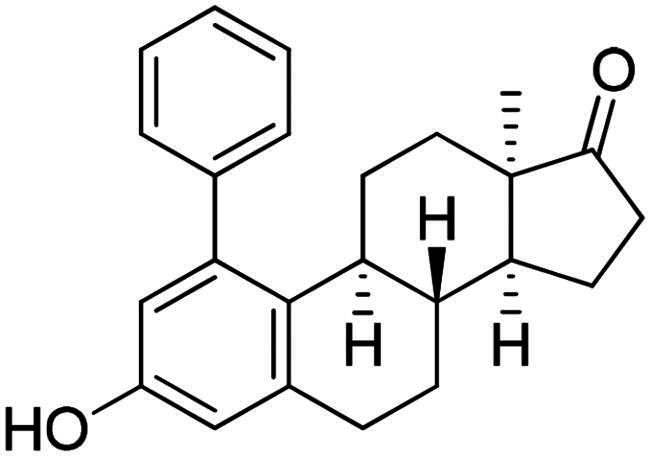	10	36.1 ± 10.2
		100	73.3 ± 7.5
**13b**	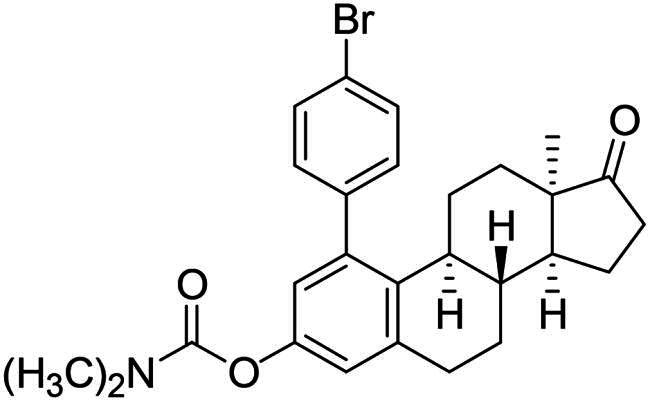	10	14.5 ± 8.4
		100	42.2 ± 18.6
**14b**	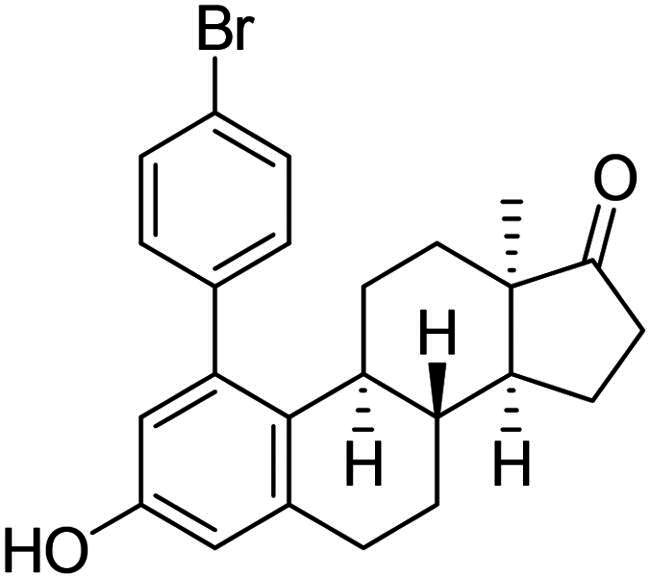	10	35.9 ± 8.0
		100	76.6 ± 5.1
**13c**	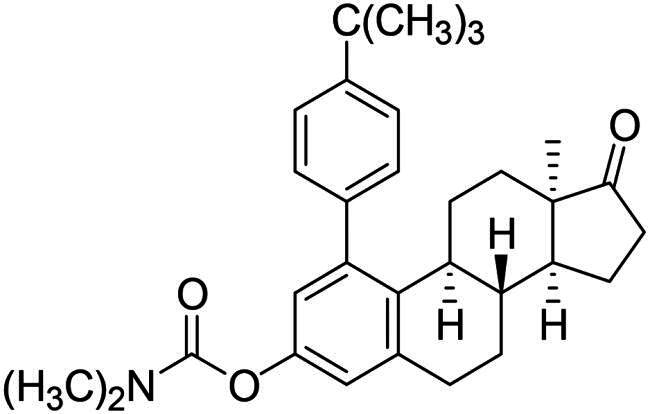	10	No inhibition
		100	No inhibition
**14c**	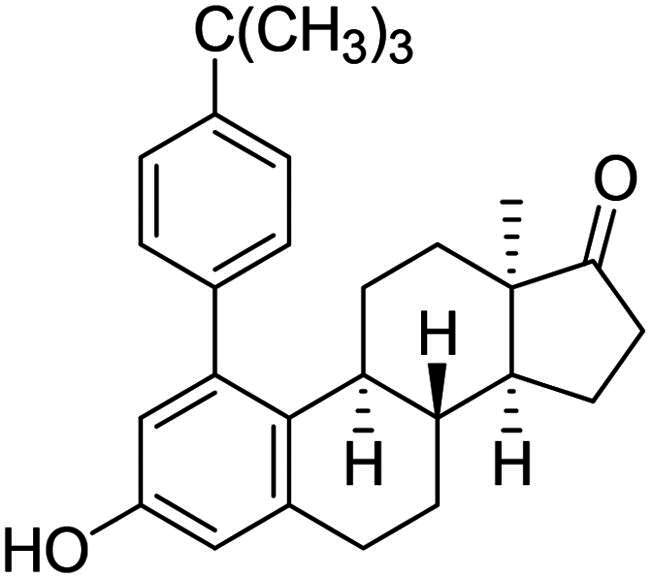	10	71.6 ± 3.2
		100	77.6 ± 6.3
		IC_50_	2.7 (1.5–4.4)
**2-Ph**	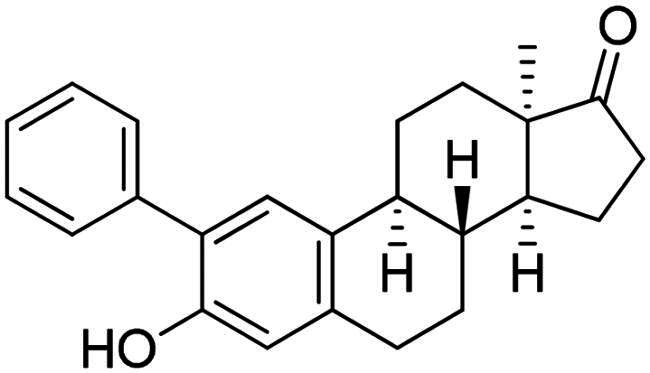	10	54.0 ± 7.8
		100	59.4 ± 7.8
**4-Ph**	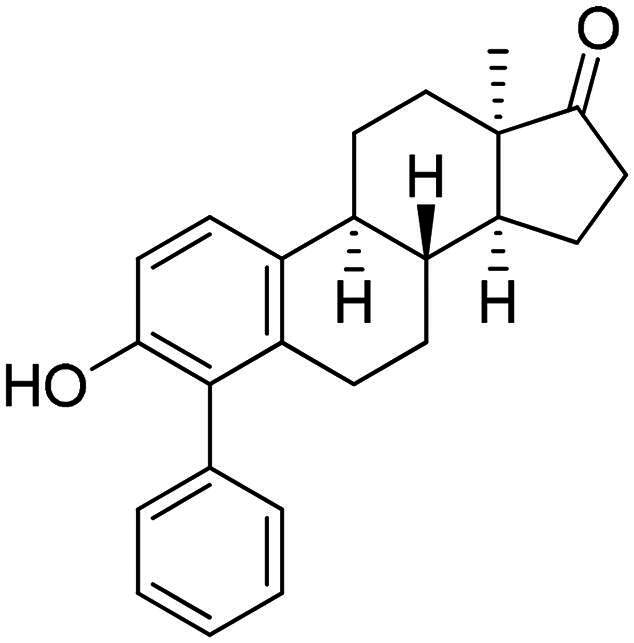	10	41.4 ± 0.2
		100	52.1 ± 17.4
**Equilin**	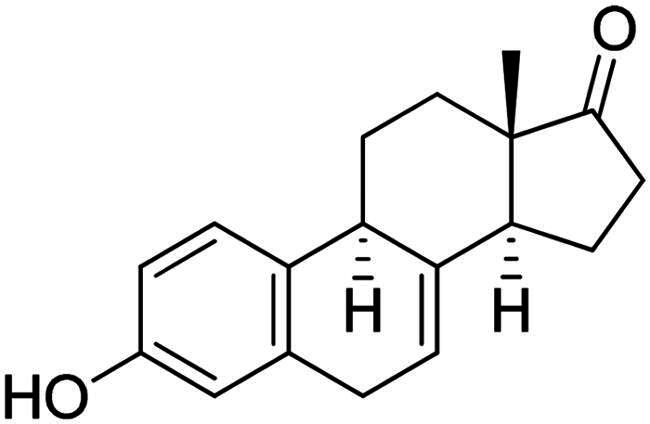	10	85.9 ± 1.6

To our great delight, *O*-arylations of protoflavone **10** led to compounds with increased anti-tumour potential. The new protoflavones **17a**–**c** displayed lower IC_50_ values (ranging from 0.39 to 1.29 µM) on cancer cell lines than compound **10** (Table 1). Interestingly, our results indicate that compounds **17a**–**c** have more pronounced activities on ER- and HPV-negative cell lines, in contrast to the data obtained here for the steroidal test compounds. The most significant submicromolar activities were observed against the HPV-negative C33A cervical cancer cell line. To the best of our knowledge, there are no literature reports on the HPV status-dependent action of protoflavones on cervical cancer cells. Although the alterations are generally not significant, the trend is obvious. Results clearly show that the data for HPV-positive and -negative cell lines do differ, sometimes by a factor of almost three. Furthermore, the same tendency can be found in the case of ER positive and negative cells (MCF-7 and MDA-MB-231): all compounds **17a**–**c** are more potent on TNBC cells with submicromolar IC_50_ values. Consequently, arylation at C-7-*O* of the protoflavone core strengthens the cytotoxic effect. Introduction of the 4-*tert*-butylphenyl group in compound **17c** was the most advantageous, with the lowest IC_50_ values on all investigated cell lines (IC_50_ = 0.39–0.64 µM), representing 2.5–5 times stronger activity than parent compound **10**. Therefore, we have chosen compound **17c** for further pharmacological experiments.

Additionally, IC_50_ values obtained for the intact NIH/3T3 mouse fibroblast cell line were higher for all protoflavones **17a**–**c** than those calculated for the cancer cell lines (1.15–5 times higher IC_50_ values). This result indicates that the investigated compounds are selective to cancerous cells.

**Figure 3.**
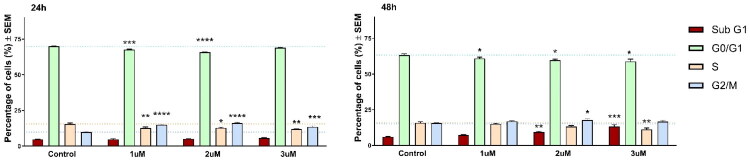
Compound **17c** induced cell cycle changes in HeLa cells after 24 and 48 h of incubation by flow cytometry with propidium iodide. The results are mean ± SEM of the data of two separate experiments performed in triplicates. *, **, and *** indicate *p* < 0.05, *p* < 0.01, and *p* < 0.001, respectively, compared to untreated control cells. Non-significant changes are not indicated.

The flow cytometry analysis revealed that the most potent compound **17c** may exert significant changes during the cell cycle, which is concentration-dependent, and can be detected after 24 and 48 h (Figure 3). Specifically, **17c** significantly increases the proportion of the cells at G2/M phase, followed by consequential reduction at S and G1 phases. In addition, substantial subG1 phase elevation occurred, which is widely interpreted as a hallmark of apoptosis. It is well-known that progressive DNA loss and reduced DNA stability occurring during apoptosis results in leakage of the low molecular weight DNA products, manifesting in a new subpopulation below the G1 phase in flow cytometry.

**Figure 4.**
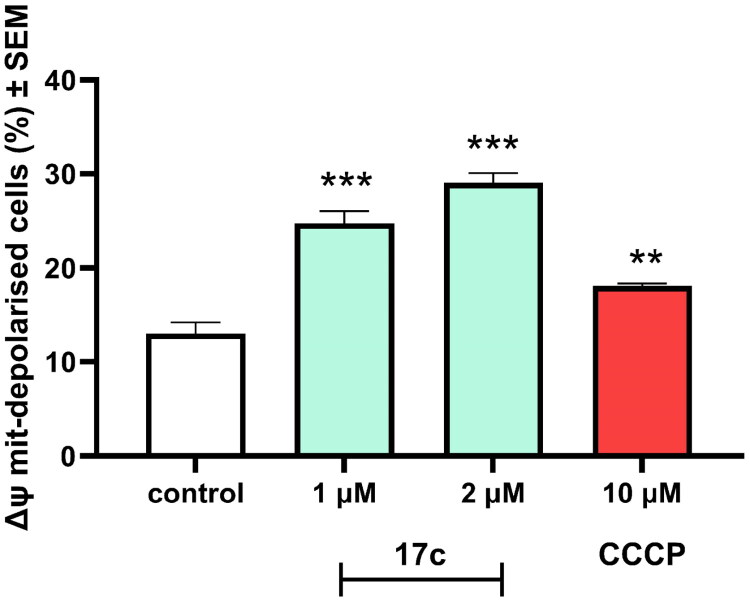
Compound **17c** induced mitochondrial membrane potential changes in Hela cells after 24 h of incubation by flow cytometry after JC-10 dye. Results are mean ± SEM of the data of two separate experiments performed in duplicates. ** and *** indicate *p* < 0.01 and *p* < 0.001, respectively, compared to untreated control cells.

During apoptosis, three functionally different stages can be seen: the initiation, effector, and degradation phases. The detectable morphological changes, such as the previously described DNA fragmentation, appear only in the final phase. However, mitochondria and mitochondrial changes are crucial during the initial stage of apoptosis. The state of the mitochondrial membrane potential is one of the main parameters of mitochondrial function. Therefore, any changes in the physiological potential can be fatal for the cells by inducing apoptosis. During our pharmacological experiments we used JC-10 dye to investigate the mitochondrial membrane potential state of the cells after **17c** incubation on HeLa cells (Figure 4). We have found that in 1 and 2 µM test concentrations, our compound significantly increased the number of mitochondrial membrane-disrupted cells after 24 h of incubation compared to the untreated controls. In addition, the effect of **17c** was more substantial than that of the applied reference agent CCCP.

These results lead to the conclusion that the investigated compound has a pronounced mitochondrial membrane disrupting effect after 24 h that triggers the initiation phase of apoptosis, which will lead to the elevated subG1 phase proportion after 48 h. Additionally, we have found that **17c** causes G2/M arrest during the cell cycle, suggesting that the cells cannot go through the mitotic procedure properly.

## Conclusions

Site-selective arylations of two nature-inspired, phenolic compounds **1** or **10** with different substituted patterns were performed with diaryliodonium salts under Cu(II) catalysis. The steroid derivative with a single phenolic OH group **1** reacted in different manners, depending on the presence of the carbamate directing group. The C—H activation and the substitution of the carbamate occurred at the *meta* position. However, the free phenolic OH group directed the aryl substitution to the sterically less hindered *ortho* position. In the case of the 1′-*O*-butyl protoapigenone **10** bearing two phenolic OH functions in the *meta* positions, O—H activation occurred, leading to 5-*O*-arylated products. We assume that the increased reactivity of 5-OH is due to the formation of an intermediate that is complexed with the copper catalyst to form a chelate. The special chemical behaviour of the two differently substituted natural compounds under the same reaction conditions is of particular novelty value. We are the first to publish the selective postfunctionalization of protoflavones on 5-OH. The methodology that we have developed to selectively transform the otherwise less reactive hydroxy group of protoflavones *via* Cu(II)-catalysis, could be extended to other flavonoids, including other types of substitution reactions.

The cytotoxic activities of the newly synthesised arylated compounds were studied against cervical or breast cancer cell lines with different HPV or hormone receptor status. The *meta*-aryl 13α-oestrone derivatives **13a**–**c**, **14a**–**c** and **16** exerted less potent cytotoxic action than the protoflavones **17a**–**c**. Introduction of the 4-*tert*-butylphenyl moiety was the most advantageous in both series. It should be emphasised that steroids and protoflavones had opposite selectivity on cell lines with different receptorial or HPV status. The submicromolar cytotoxic potential of the 5-*O*-arylated protoflavones against TNBC and HPV-negative cervical cancer cells is a new finding and a promising starting point for the development of novel, selective drug candidates. The 17β-HSD1 enzyme inhibitory study was performed for the newly synthesised *meta*-arylated 13α-oestrone derivatives **13a**–**c**, **14a**–**c**. Compounds bearing free hydroxy group **14a**–**c** proved to be more potent than their carbamate counterparts **13a**–**c**. As the most active inhibitor, the *tert*-butylphenyl derivative **14c** displayed IC_50_ value in a low micromolar range. To the best of our knowledge, this is the first evidence in the literature for the 17β-HSD1 inhibitory activity of 1-arylated 13α-oestrone derivatives.

## Supplementary Material

Supporting_23_05- Clean.docx

## Data Availability

The authors confirm that the data supporting the findings of this study are available within the article and its supplementary materials.
