# Supplementary material for: Site-selective arylations of nature-inspired flavonoids or steroidal phenols via C—H or O—H activation
Source: J Enzyme Inhib Med Chem. 2025 Jul 18;40(1):2530615. doi: 10.1080/14756366.2025.2530615 (PMC12278462; doi:10.1080/14756366.2025.2530615)
Supplement: Supporting_23_05- Clean.docx [file IENZ_A_2530615_SM2091.docx]

**1. Experimental**

**1.1. Chemistry**

Melting points (Mp) were determined with Kofler hot-stage apparatus and are uncorrected. Thin-layer chromatography was performed on silica gel 60 F254 (layer thickness 0.2 mm, Merck); eluent: 35% ethyl acetate/65% hexanes. The spots were detected with I2 or UV (365 nm) after spraying with 5% phosphomolybdic acid in 50% aqueous phosphoric acid and heating at 100–120 °C for 10 min. Column chromatography was performed on silica gel 60, 40–63 μm (Merck). ^1^H NMR spectra were recorded in DMSO-d_6_ or CDCl_3_ solution with a Bruker DRX-500 instrument at 500 MHz. ^13^C NMR spectra were recorded with the same instrument at 125 MHz under the same conditions. Mass spectrometry: full scan mass spectra of the newly synthesized compounds were acquired in the range of 100 to 1100 m/z with a Q Exactive Plus quadrupole-orbitrap mass spectrometer (Thermo Fisher Scientific, Waltham, MA, USA) equipped with a heated electrospray (HESI). Analyses were performed in positive or negative ion mode by flow injection mass spectrometry with a mobile phase of 50% aqueous acetonitrile containing 0.1 v/v% formic acid (0.3 ml min−1 flow rate). Aliquots of 5 µl of samples were injected into the flow. The ESI capillary was adjusted to 3.5 kV and N_2_ was used as a nebulizer gas.

*General procedure A: meta- or O-arylation*

The 13α-estrone carbamate (**12**, 0.50 mmol) or the protoflavone (**10**, 0.50 mmol) was dissolved in 1,2-dichloroethane (10.0 mL) and the appropriate iodonium triflate (0.60 mmol) and Cu(OTf)_2_ (18 mg, 0.050 mmol) were added. The reaction was stirred at 70 °C for the 3 h. After the dilution with CH_2_Cl_2_ (100 mL), the mixture was washed with saturated sodium bicarbonate solution (50 mL). The aqueous phase was extracted with CH_2_Cl_2_ (3x15 mL), and the combined organic phases were dried over sodium sulphate and evaporated in vacuo. The crude product was purified by column chromatography.

*1.1.1. 3-(N,N-Dimethylcarbamoyloxy)-1-phenyl-13α-estra-1,3,5(10)-triene-17-one (****13a****) and 3-hydroxy-1-phenyl-13α-estra-1,3,5(10)-triene-17-one (****14a****)*

As described in General procedure A, carbamate **12** (170.6 mg) was reacted. Purification by column chromatography (gradient elution) using hexanes/*tert*-Butyl methyl ether (MTBE) from 9:1 (v/v) to 7:3 (v/v) as eluent afforded compound **13a** and **14a.** The first-eluted compound **14a** was obtained as a white solid (40 mg, 23 %). M.p.: 226–228 °C, R_f_ = 0.51. ^1^H-NMR (DMSO-d_6_) δ (ppm): 0.85 (s, 3H, 18-H_3_); 6.38 (d, 1H, *J*= 2.2 Hz, 4-H); 6.44 (d, 1H, *J*= 2.2 Hz, 2-H); 7.30—7.33 (overlapping multiplets, 3H); 7.40 (m, 2H); 9.11 (s, 1H, OH). ^13^C-NMR (CDCl_3_) δ (ppm): 20.7 (CH_2_); 24.9 (C-18); 26.8 (CH_2_); 29.9 (CH_2_); 32.2 (CH_2_); 32.7 (CH_2_); 33.4 (CH_2_); 41.8 (CH); 44.0 (CH); 49.9 (CH); 50.2 (C-13); 114.4 (C-2); 116.6 (C-4); 126.8 (C-4’); 128.0 and 128.4 (C-2’, -3’, -5’ and -6’); 129.8 (C); 140.2 (C); 143.7 (C); 144.0 (C); 152.9 (C); 221.7 (C-17). ESI-HRMS: m/z: 391.19145 [M + HCOO]^-^ (C_24_H_26_O_2_ + HCOO^-^ requires 391.19094). Continued elution yielded compound **13a** as a white solid (120 mg, 58 %). M.p.: 228–229 °C, R_f_ = 0.37. ^1^H-NMR (CDCl_3_) δ (ppm): 0.93 (s, 3H, 18-H_3_); 2.86 (m, 2H, 6-H_2_); 2.99 and 3.06 (2xs, 2x3H, N(CH_3_)_2_); 6.78 (d, 1H, *J*= 2.2 Hz, 4-H); 6.84 (d, 1H, *J*= 2.2 Hz, 2-H); 7.29 (t, 1H, *J* = 7.3 Hz, 4’-H); 7.33—7.38 (overlapping multiplets, 4H). ^13^C-NMR δ (ppm): 20.7 (CH_2_); 24.9 (C-18); 26.7 (CH_2_); 29.7 (CH_2_); 32.2 (CH_2_); 32.8 (CH_2_); 33.4 (CH_2_); 36.4 and 36.7 (N(CH_3_)_2_); 42.0 (CH); 43.9 (CH); 49.9 (CH); 50.2 (C-13); 121.0 (C-2); 122.4 (C-4); 126.8 (C-4’); 128.0 (2C) and 128.4 (2C): C-2’, -3’, -5’ and -6’; 134.3 (C); 139.7 (C); 143.4 (C); 143.6 (C); 148.9 (C); 155.1 (C); 221.5 (C=O). ESI-HRMS: m/z: 418.23782 [M + H]^+^ (C_27_H_31_NO_3_ + H^+^ requires 418.23822).

*1.1.2. 1-(4-bromophenyl)-3-(N,N-dimethylcarbamoyloxy)-13α-estra-1,3,5(10)-triene-17-one (****13b****) and 1-(4-bromophenyl)-3-hydroxy-13α-estra-1,3,5(10)-triene-17-one (****14b****)*

As described in General procedure A, carbamate **12** (170.6 mg) was reacted. Purification by column chromatography (gradient elution) using hexanes/*tert*-Butyl methyl ether (MTBE) from 9:1 (v/v) to 7:3 (v/v) as eluent afforded compound **13b** and **14b.** The first-eluted compound **14b** was isolated as white crystals (60 mg, 28 %). M.p.: 259–260 °C, R_f_ = 0.47. ^1^H-NMR (DMSO-d_6_) δ (ppm): 0.87 (s, 3H, 18-H_3_); 2.66 (m, 2H, 6-H_2_); 6.36 (d, 1H, *J*= 2.2 Hz, 4-H); 6.46 (d, 1H, *J*= 2.2 Hz, 2-H); 7.29 (d, 2H, *J* = 8.3 Hz) and 7.58 (d, 2H, *J* = 8.3 Hz): 2’-, 3’-, 5’- and 6’-H; 9.16 (OH). ^13^C-NMR δ (ppm): 20.0 (CH_2_); 24.2 (C-18); 26.1 (CH_2_); 29.8 (CH_2_); 31.4 (CH_2_); 32.2 (CH_2_); 32.6 (CH_2_); 40.5 (CH); 43.1 (CH); 48.9 (CH); 49.4 (C-13); 114.5 (C-2); 116.0 (C-4); 119.6 (C); 127.4 (C); 129.7 and 131.2: C-2’, -3’, -5’ and -6’; 139.7 (C); 141.3 (C); 142.9 (C); 154.5 (C); 220.4 (C). ESI-HRMS: m/z: 469.10223 [M + HCOO]^-^ (C_24_H_25_BrO_2_ + HCOO^-^ requires 469.10145). Continued elution yielded compound **13b** as white crystals (180 mg, 72 %). M.p.: 111–113 °C, R_f_ = 0.37. ^1^H-NMR (CDCl_3_) δ (ppm): 0.95 (s, 3H, 18-H_3_); 2.85 (m, 2H, 6-H_2_); 2.99 and 3.06 (2xs, 2x3H, N(CH_3_)_2_); 6.74 (d, 1H, *J*= 2.2 Hz, 4-H); 6.85 (d, 1H, *J*= 2.2 Hz, 2-H); 7.22 (d, 2H, *J* = 8.3 Hz) and 7.50 (d, 2H, *J* = 8.3 Hz): 2’-, 3’-, 5’- and 6’-H. ^13^C-NMR δ (ppm): 20.7 (CH_2_); 24.9 (C-18); 26.6 (CH_2_); 29.8 (CH_2_); 32.1 (CH_2_); 32.8 (CH_2_); 33.3 (CH_2_); 36.4 and 36.7 (N(CH_3_)_2_); 42.0 (CH); 43.9 (CH); 49.8 (CH); 50.2 (C-13); 120.9 (C-4’); 121.4 (C-2); 122.4 (C-4); 129.7 and 131.6 (C-2’, -3’, -5’ and -6’); 134.2 (C); 140.0 (C); 142.2 (C); 142.4 (C); 149.0 (C); 155.0 (C); 221.3 (C=O). ESI-HRMS: m/z: 496.14927 [M + H]^+^ (C_27_H_30_BrNO_3_ + H^+^ requires 496.14873).

*1.1.3. 3-(N,N-dimethylcarbamoyloxy)-1-(4-tert-butylphenyl)-13α-estra-1,3,5(10)-triene-17-one (****13c****) and 3-hydroxy-1-(4-tert-butylphenyl)-13α-estra-1,3,5(10)-triene-17-one (****14c****)*

As described in General procedure A, carbamate **12** (170.6 mg) was reacted. Purification by column chromatography (gradient elution) using hexanes/*tert*-Butyl methyl ether (MTBE) from 9:1 (v/v) to 7:3 (v/v) as eluent afforded compound **13c** and **14c.** The first-eluted compound **14c** was isolated as white crystals (57 mg, 28 %). M.p.: 217–219 °C, R_f_ = 0.55. ^1^H-NMR (DMSO-d_6_) δ (ppm): 0.85 (s, 3H, 18-H_3_); 1.31 (s, 9H, C(CH_3_)_3_); 6.38 (d, 1H, *J*= 2.2 Hz, 4-H); 6.42 (d, 1H, *J*= 2.2 Hz, 2-H); 7.23 (d, 2H, *J* = 8.3 Hz) and 7.40 (d, 2H, *J* = 8.3 Hz): 2’-, 3’-, 5’- and 6’-H; 9.09 (s, 1H, OH). ^13^C-NMR δ (ppm): 20.7 (CH_2_); 24.9 (C-18); 26.8 (CH_2_); 30.1 (CH_2_); 31.7 (3C, C(CH_3_)_3_); 32.1 (CH_2_); 32.7 (CH_2_); 33.3 (CH_2_); 34.7 (C(CH_3_)_3_); 41.5 (CH); 43.8 (CH); 49.6 (CH); 50.0 (C-13); 114.6 (C-2); 116.6 (C-4); 125.5 and 127.8: C-2’, -3’, -5’ and -6’; 128.2 (C-4’); 140.0 (C); 141.3 (C); 143.2 (C); 149.5 (C); 155.0 (C); 221.1 (C-17). ESI-HRMS: m/z: 447.25372 [M + HCOO]^-^ (C_28_H_34_O_2_ + HCOO^-^ requires 447.25354). Continued elution yielded compound **13c** as white crystals (171 mg, 72 %). M.p.: 168–169 °C, R_f_ = 0.44. ^1^H-NMR (CDCl_3_) δ (ppm): 0.94 (s, 3H, 18-H_3_); 1.35 (s, 9H, C(CH_3_)_3_); 2.85 (m, 2H, 6-H_2_); 2.99 and 3.06 (2xs, 2x3H, N(CH_3_)_2_); 6.79 (d, 1H, *J*= 2.2 Hz, 4-H); 6.83 (d, 1H, *J*= 2.2 Hz, 2-H); 7.25 (d, 2H, *J* = 8.3 Hz) and 7.37 (d, 2H, *J* = 8.3 Hz): 2’-, 3’-, 5’- and 6’-H. ^13^C-NMR δ (ppm): 20.8 (CH_2_); 25.0 (C-18); 26.8 (CH_2_); 29.5 (CH_2_); 31.4 (3C, C(CH_3_)_3_); 32.2 (CH_2_); 32.7 (CH_2_); 33.4 (CH_2_); 34.5 (C(CH_3_)_3_); 36.4 and 36.7 (N(CH_3_)_2_); 42.1 (CH); 43.9 (CH); 49.9 (CH); 50.2 (C-13); 120.8 (C-2); 122.3 (C-4); 125.2 and 127.6: C-2’, -3’, -5’ and -6’; 134.3 (C); 139.6 (C); 140.4 (C); 143.5 (C); 148.9 (C); 149.8 (C); 155.0 (C); 221.6 (C=O). ESI-HRMS: m/z: 474.30059 [M + H]^+^ (C_31_H_39_NO_3_ + H^+^ requires 474.30082).

*1.1.4. 3-[{1-Benzyl-1H-1,2,3-triazol-4-yl}methoxy]-1-(4-bromophenyl)-13a-estra-1,3,5 (10)-trien-17-one (****16****)*

1-(4-Bromophenyl)-3-hydroxy-13α-estra-1,3,5(10)-triene-17-one (**14b**, 127 mg, 0.3 mmol) was dissolved in acetone (5 mL), and propargyl bromide (0.05 ml (80 wt.% in toluene), 0.45 mmol), K_2_CO_3_ (277 mg, 2 mmol) and 18-crown-6 (8 mg, 0.03 mmol) were added. The reaction mixture was stirred at 70 °C for 3 h, the solvent was then evaporated off. The residue was dissolved in dichlorometane, and the solution was washed with water until neutral. The dichloromethane solution was dried over sodium sulphate and evaporated in vacuo. The crude product was dissolved in toluene (3 mL), Ph_3_P (8 mg, 0.03 mmol), CuI (3 mg, 0.015 mmol), DIPEA (0.16 ml, 1.0 mmol) and benzylazide (40 mg, 0.3 mmol) were added. The reaction mixture was refluxed for 2h, then allowed to cool and evaporated in vacuo. Purification by column chromatography using hexanes/ethyl acetate 3:7 (v/v) as eluent afforded compound **16** as a white solid (152 mg, 85 %). M.p.: 215–217 °C, R_f_ = 0.21. ^1^H-NMR (DMSO-d_6_) δ (ppm): 0.87 (s, 3H, 18-H_3_); 2.66 (m, 2H, 6-H_2_); 5.10 (m, 2H); 5.59 (s, 2H); 6.60 (d, 1H, *J*= 2.2 Hz, 4-H); 6.75 (d, 1H, *J*= 2.2 Hz, 2-H); 7.30–7.38 (overlapping multiplets, 7H, 2’-, 2”-, 3”-, 4”-, 5”-, 6’- and 6”-H); 7.6 (d, 2H, *J* = 8.1 Hz, 3’- and 5’-H); 8.22 (C=CH). ^13^C-NMR δ (ppm): 20.0 (CH_2_); 24.2 (C-18); 25.9 (CH_2_); 29.6 (CH_2_); 31.5 (CH_2_); 32.2 (CH_2_); 32.6 (CH_2_); 40.5 (CH); 43.0 (CH); 48.9 (CH); 49.4 (C-13); 52.7 (NCH_2_); 60.9 (OCH_2_); 113.9 (C-2); 115.5 (C-4); 119.9 (C-4’); 124.3 (C=CH); 127.8 (2C, 2xCH); 128.0 (CH); 128.6 (2C, 2xCH); 129.5 (C); 129.8 (2C, 2xCH); 131.2 (2C, 2xCH); 135.8 (C); 140.0 (C); 141.4 (C); 142.5 (C); 143.0 (C); 155.3 (C); 220.3 (C).

*1.1.5. 2-(1-Butoxy-4-oxocyclohexa-2,5-dienyl)-7-hydroxy-5-phenoxy-chromen-4-one (****17a****)*

As described in General procedure A, protoflavone **10** (171 mg) was reacted. Purification by column chromatography, using hexanes/EtOAc = 7:3 (v/v) as eluent afforded compound **17a**, which was isolated as yellow crystals (178 mg, 85 %). M.p.: 212–214 °C, R_f_ = 0.20. ^1^H-NMR (DMSO-d_6_) δ (ppm): 0.90 (t, 3H, *J* = 7.4 Hz, Bu-CH_3_); 1.38 (m, 2H, Bu-CH_2_); 1.56 (m, 2H, Bu-CH_2_); 3.47 (t, 2H, *J* = 6.3 Hz, OCH_2_); 6.21 (d, 1H, *J* = 1.8 Hz) and 6.43 (d, 1H, *J* = 1.8 Hz): 6-H and 8-H; 6.27 (s, 1H, 3-H); 6.51 (d, 2H, *J* = 10.0 Hz, 2’-H and 6’–H); 6.95 (d, 2H, *J* = 7.7 Hz, 2”-H and 6”–H); 7.08 (d, 2H, *J* = 10.0 Hz, 3’-H and 5’–H); 7.13 (t, 1H, *J* = 7.7 Hz, 4”-H); 7.37 (d, 2H, *J* = 7.7 Hz, 3”-H and 5”–H); 10.92 (s, 1H, 7-OH). ^13^C-NMR δ (ppm): 13.5 (Bu-CH_3_); 18.6 (CH_2_); 31.4 (CH_2_); 64.0 (OCH_2_); 73.5 (C-1’); 98.2 (CH); 104.5 (CH); 108.9 (C); 109.6 (CH); 118.3 (2C, 2xCH); 123.3 (C-4”); 129.8 (2C, 2xCH); 131.7 (2C, 2xCH); 146.4 (2C, 2xCH); 156.4 (C); 156.8 (C); 158.7 (C); 161.4 (C); 162.6 (C); 174.3 and 184.2 (C-4 and C-4’). ESI-HRMS: m/z: 419.14901 [M + H]^+^ (C_25_H_22_O_6_ + H^+^ requires 419.14947).

*1.1.6. 5-(4-Bromophenoxy)-2-(1-butoxy-4-oxocyclohexa-2,5-dienyl)-7-hydroxy-chromen-4-one (****17b****)*

As described in General procedure A, protoflavone **10** (171 mg) was reacted. Purification by column chromatography, using hexanes/EtOAc = 7:3 (v/v) as eluent afforded compound **17b**, which was isolated as yellow crystals (219 mg, 88 %). M.p.: 108–110 °C, R_f_ = 0.30. ^1^H-NMR (DMSO-d_6_) δ (ppm): 0.89 (t, 3H, *J* = 7.4 Hz, Bu-CH_3_); 1.37 (m, 2H, Bu-CH_2_); 1.56 (m, 2H, Bu-CH_2_); 3.47 (t, 2H, *J* = 6.3 Hz, OCH_2_); 6.27 (s, 1H, 3-H); 6.34 (d, 1H, *J* = 1.8 Hz); 6.51—6.53 (overlapping multiplets, 3H); 6.86 (d, 2H, *J* = 8.7 Hz, 2”-H and 6”–H); 7.07 (d, 2H, *J* = 10.0 Hz, 3’-H and 5’–H); 7.49 (d, 1H, *J* = 8.7 Hz, 3”-H and 5”–H); 10.94 (s, 1H, 7-OH). ^13^C-NMR δ (ppm): 13.5 (Bu-CH_3_); 18.6 (CH_2_); 31.4 (CH_2_); 64.1 (OCH_2_); 73.5 (C-1’); 99.1 (CH); 105.9 (CH); 109.3 (C); 109.6 (CH); 114.5 (C-4”); 119.6 (2C, 2xCH); 131.8 (2C, 2xCH); 132.4 (2C, 2xCH); 146.3 (2C, 2xCH); 155.5 (C); 156.4 (C); 158.7 (C); 161.7 (C); 162.6 (C); 174.2 and 184.2 (C-4 and C-4’). ESI-HRMS: m/z: 497.06003 [M + H]^+^ (C_25_H_21_BrO_6_ + H^+^ requires 497.05998).

1.1.7. 2-(1-Butoxy-4-oxocyclohexa-2,5-dienyl)-5-(4-*tert*-butylphenoxy)-7-hydroxy-chromen-4-one (**17c**)

As described in General procedure A, protoflavone **10** (171 mg) was reacted. Purification by column chromatography, using hexanes/EtOAc = 7:3 (v/v) as eluent afforded compound **17c**, which was isolated as yellow crystals (204 mg, 86 %). M.p.: 219–221 °C, R_f_ = 0.32. ^1^H-NMR (DMSO-d_6_) δ (ppm): 0.89 (t, 3H, *J* = 7.4 Hz, Bu-CH_3_); 1.28 (s, 9H, C(CH_3_)_3_); 1.39 (m, 2H, 3’’’-CH_2_); 1.56 (m, 2H, 2’’’-CH_2_); 3.47 (t, 2H, *J* = 6.3 Hz, OCH_2_); 6.17 (d, 1H, *J* = 1.8 Hz) and 6.38 (d, 1H, *J* = 1.8 Hz): 6-H and 8-H; 6.27 (s, 1H, 3-H); 6.51 (d, 2H, *J* = 10.0 Hz, 2’-H and 6’–H); 6.86 (d, 2H, *J* = 8.5 Hz, 2”-H and 6”–H); 7.08 (d, 2H, *J* = 10.0 Hz, 3’-H and 5’–H); 7.38 (d, 1H, *J* = 8.5 Hz, 3”-H and 5”–H). ^13^C-NMR δ (ppm): 13.5 (Bu-CH_3_); 18.6 (3’’’-CH_2_); 31.1 (C(CH_3_)_3_); 31.4 (2’’’-CH_2_); 33.9 (C(CH_3_)_3_); 64.0 (OCH_2_); 73.5 (C-1’); 98.0 and 104.0 (2xCH, C-6 and C-8); 108.6 (C-4a); 109.6 (C-3); 118.0 (2C, C-2” and C-6”); 126.4 (2C, C-3” and C-5”); 131.7 (2C, C-2’ and C-6’); 145.7 (C-4”); 146.3 (2C, C-3’ and C-5’); 153.9 (C-1”); 157.3 (C-8a); 158.7 and 162.8 (2xC, C-6 and C-8); 161.3 (C-2); 174.4 (C-4); 184.2 (C-4’). ESI-HRMS: m/z: 475.21223 [M + H]^+^ (C_29_H_30_O_6_ + H^+^ requires 475.21207).

*General procedure B: hydrolysis of meta-arylated carbamates*

The carbamate (**13a**–**c**, 0.2 mmol) was dissolved in isopropyl alcohol (3 mL) and 6 equivalents of NaOH was added. The reaction mixture was heated at 80 °C for 2 h. After cooling to rt, solvent was evaporated and the residual was acidified with 1 M HCl solution. After extraction with ethyl acetate (3 x 15 mL), the combined organic layers were dried, evaporated and purified by column chromatography using hexanes/EtOAc = 8:2 (v/v) as eluent.

Compound **14a** was isolated in 93 % yield (65 mg).

Compound **14b** was isolated in 94 % yield (93 mg).

Compound **14c** was isolated in 90 % yield (85 mg).

*Procedure C: ortho-arylation*

The 13α-estrone (**1**, 0.50 mmol) was dissolved in 1,2-dichloroethane (10.0 mL) and diphenyliodonium triflate (0.60 mmol) and Cu(OTf)_2_ (18 mg, 0.050 mmol) were added. The reaction was stirred at 70 °C for the 3 h. After the dilution with CH_2_Cl_2_ (100 mL), the solution was washed with saturated sodium bicarbonate solution (50 mL). The aqueous phase was extracted with CH_2_Cl_2_ (3x15 mL), and the combined organic phases were dried over magnesium sulphate and evaporated in vacuo. The crude product was purified by column chromatography using hexanes/EtOAc = 8:2 (v/v) as eluent. Compound **2a** was isolated as white crystals (156 mg, 90 %). Compound **2a** was identical with compound described in the literature^6^. M.p.: 200–203 °C, R_f_ = 0.50. 1H NMR (DMSO‑d6) δ ppm: 0.95 (s, 3H, H-18), 2.71 (m, 2H, H-6), 6.60 (s, 1H, H-4), 7.08 (s, 1H, H-1), 7.25 (t, J =7.5 Hz, 1H, H-4′), 7.34 (t, J =7.5 Hz, 2H, H-3′and H-5′), 7.50 (d, J =7.1 Hz, 2H, H-2′and H-6′), 9.17 (s, 1H, 3-OH).

**1.2. Spectroscopic measurements**

Stock solutions of compounds **10** and **17b** were prepared in methanol (0.05 mg/mL each). Absorption spectra were recorded applying a BMG Labtech Spectro Star Nano UV-Vis spectrophotometer (Ortenberg, Germany) at 25 °C. Thereafter, 40 μL of AlCl_3_ solution (1 g AlCl_3_.6H_2_O was dissolved in 20 mL of methanol) was added into 100 μL of stock solutions of compounds **10** or **17b**, and the absorption spectra were recorded again (3 min after addition). The spectra were displayed using the instrument software.

**1.3. Pharmacology**

***Antiproliferative assay***

Antiproliferative screening and determination of IC_50_ values of the active compounds were measured by standard MTT assay, after 72 hours incubation.

Briefly, the panels of breast and cervical cancerous cell lines, differing by their receptorial and HPV status were used, precisely MCF-7 (ER, PR and HER2 positive) and MDA-MB-231 (TNBC) breast carcinoma and HeLa (HPV18+), SiHa (HPV16+), C33A (HPV-) cervical carcinoma cells. Non-cancerous NIH/3T3 mouse fibroblast cell line was used for selectivity study. MCF‑7, MDA-MB-231, Hela and NIH/3T3 cell lines were purchased from ECACC (European Collection of Cell Cultures, Salisbury, UK), while SiHa and C33A from ATCC (American Tissue Culture Collection, LGC Standards GmbH, Wesel, Germany). Cells were maintained in Eagle's Minimum Essential Medium (EMEM, Capricorn Scientific GmbH, Ebsdorfergrund, Germany) supplemented with 10% heat-inactivated fetal bovine serum (FBS), 1% non-essential amino acids (NEAA), and 1% antibiotic-antimycotic mixture (penicillin–streptomycin). The cells were maintained at 37 °C in a humidified atmosphere containing 5% CO_2_.

For measurements cells were seeded for 96-well plates at a density of 5 000/well, except for C33A, which was seeded in 10 000cells for each well. After overnight standing cells were treated with 10 and 30 µM solutions for screening and increasing concentrations (0.1‑30.0 µM) of the test compounds were used for concentration-response curves. After incubation for 72 hours under cell culturing condition 5 mg/ml MTT (3-(4,5-dimethyl- azole-2-yl)-2,5-diphenyl-2H-tetrazolium bromide) solution was added for another 4 h. The precipitated formazan crystals were solubilized in DMSO and the absorbance was measured at 545 nm with a microplate reader (BMG Labtech, Ortenberg, Germany). Wells with untreated cells were utilized as control, and inhibition % were calculated by GraphPad Prism v 9.0 software (GraphPad Software, San Diego, CA, USA). If the growth-inhibition exceeded 70% at 30 µM concentration during the screening concentration-response curves were fitted and IC_50_ values were calculated by GraphPad Prism v 9.0. The experiments were performed with five parallel wells for each experimental condition and repeated at least twice.

***Cell cycle analysis by flow cytometry***

To determine the cellular DNA content of the cells, flow cytometry analysis after propidium-iodide labeling was performed. Propidium iodide is a nuclear dye intercalating between the base pairs in DNA without sequence preference. Therefore, the cells' total emitted fluorescence will depend on each cell's DNA content, which varies depending on the cell cycle phase.

Hela cells were seeded into 12-well plates at 200,000 cells/well density for the cell cycle analysis. Cells were incubated overnight prior to the treatment, then treated with 1, 2, and 3 µM solutions of 14c compound for 24 h and 48 hours. After incubation, cells were washed twice with phosphate-buffered saline (PBS) and harvested. After centrifugation at 1200 rpm, for 7 minutes, cells were washed again with 300 µl PBS and fixed in 500 µl cold 70% ethanol. Samples were stained in 300 µl dye solution containing RNaseA, TritonX-100, sodium citrate, and 0.1 mg/ml-PI in distilled water. The suspension was incubated for 20 minutes in the dark at room temperature. Cells were measured by CytoFLEX flow cytometer, recorded by CytExpert Software (Beckman Coulter INC, Brea, CA, USA). In each analysis, 20,000 events were recorded inside the FSC/SSC gate, and the percentages of the cells in the different phases of the cell cycle were determined and analyzed using ModFit LT 6.0 software.

***Mitochondrial membrane potential assay***

JC-10 dye was used to investigate the changes in the mitochondrial membrane potential. JC‑10 is a cationic dye that can pass through the cell membrane and accumulate in healthy mitochondria by forming reversible red-fluorescent aggregates. In addition, changes in membrane potential result in the failure to retain the aggregates. Therefore, the dye returns to the cytosol in green monomeric form. Mitochondrial changes may be detected by measuring the green and red fluorescent signals together.

Hela cells were seeded into 12-well plates at 200,000 cells/well density and incubated overnight. Cells were treated with 1 and 2 µM of **17c** for 24 h and 10 µM of CCCP as reference compound ^S1^. After the incubation, cells were collected and centrifuged at 1100 rpm for 6 minutes, then washed with PBS. After centrifugation, 300 µl dye solution (20 µM JC-10 final concentration) was added to the pellet. After 5 min incubation, cells were measured by CytoFLEX flow cytometer, recorded by CytExpert Software (Beckman Coulter INC, Brea, CA, USA) reading of red and green fluorescence on 525 and 585 channels.

***Enzymatic assay***

The bacterial homogenate of E. coli overexpressing HSD17B1 was previously prepared as described in references ^S2,S3^, and stored in aliquots at -80 °C. Before conducting experiments, aliquots were quickly thawed and diluted twice with 100 mM sodium phosphate buffer (pH 6.5; #14457, #1411007, Kemika), then kept on ice. The enzymatic assay was performed as described in ^S3^. Briefly, a 1 mL enzymatic reaction mixture was prepared in 2 mL microcentrifuge tubes with the following components: 10 µL of the test inhibitor dissolved in dimethyl sulfoxide (DMSO; #276855, Sigma Aldrich) or 10 µL of DMSO for control samples, 5 µL of 14 µM substrate estrone (E1; CAS 53-16-7; #E2300-000, Steraloids), 10 µL of 10 mM NADPH (#AE14, Carl Roth) prepared in 100 mM sodium phosphate buffer, and 965 µL of 100 mM sodium phosphate buffer (pH 6.5). The reaction mixture was mixed thoroughly before adding 10 µL of bacterial homogenate. Immediately after addition, the tubes were transferred to an Eppendorf thermoshaker (#5382000031, Eppendorf) and incubated at 37 °C for 10 minutes. To stop the reaction, 60 µL of 1.26 M ascorbic acid (#A92902, Sigma Aldrich) in methanol (#34966, Honeywell/Riedel-de Haen) /acetic acid (#1.00063, Merck) (99:1, v/v) was added. The samples were then immediately processed using solid-phase extraction (SPE). The assay was repeated in three independent experiments, with each experiment performed in technical duplicate.

***Sample preparation for LC-MS/MS analysis***

The preparation of samples for liquid chromatography-tandem mass spectrometry (LC-MS/MS) involved SPE. Stock solutions of E1 and 17β-estradiol (E2; CAS: 50-28-2; #142406, Steraloids) were prepared gravimetrically and dissolved in methanol (#34966, Honeywell/Riedel-de Haen). A working calibrator solution containing both steroids was then prepared by mixing appropriate volumes of the stock solutions in methanol to achieve a final concentration of 1 µg/mL for each steroid. Eight-point calibration curves (excluding zero and blank) were prepared in 100 mM sodium phosphate buffer (pH 6.5). For each 1 mL reaction mixture or calibrator, 10 µL of 100 ng/mL internal standard solution containing [2,3,4-13C3]-17β-estradiol (#719552, Sigma Aldrich) was added. Samples were mixed thoroughly and incubated at room temperature for 15 minutes.

SPE was performed using Strata X 33 µm polymeric reversed-phase columns (30 mg/mL tubes; #8B-S100-TAK, Phenomenex). The procedure involved the following steps: column conditioning with 1 mL of methanol, column equilibration with 1 mL of H₂O (#1.15333, Supelco), sample loading (1 mL sample or calibrator), column washing with 1 mL of H₂O, column drying under high vacuum for 10 minutes, and finally elution with 1 mL of methanol directly into HPLC glass vials (#702713, Macherey-Nagel GmbH/Co). The eluted samples were stored at -20 °C until LC-MS/MS analysis.

***LC-MS/MS analysis***

LC-MS/MS analysis of E1 and E2 was performed as described in Pavlič et al. [4]. Chromatographic separation was carried out using a Shimadzu Nexera XR HPLC system (Shimadzu Corporation, Kyoto, Japan) equipped with a Kinetex 2.6 µm XB-C18 column (100 × 4.6 mm; #00D-4496-E0, Phenomenex). The mobile phases used were phase A: 5% methanol in H₂O with 0.2 mM ammonium fluoride (NH₄F; # 52481, Honeywell/Fluka), phase B: methanol with 0.2 mM NH₄F. A linear gradient from 30% to 96% B was applied from 1.0 to 3.0 minutes followed by a wash with 96% B until 8 minutes, and then equilibration with the starting conditions until 15 minutes. The flow rate was 0.5 mL/min, the injection volume 5 µL. The eluate was then introduced into a Sciex 3500 triple quadrupole mass spectrometer (AB Sciex Deutschland GmbH, Darmstadt, Germany), operated in negative electrospray ionization (ESI) mode. The following MS parameters were used: curtain gas: 50 psi, collision gas: 8 psi, ion spray voltage: −4500 V, source temperature: 600°C, ion source gases 1 and 2: 40 and 80 psi, respectively. Data processing and quantification were performed using Analyst 1.6 software (AB Sciex Deutschland GmbH, Darmstadt, Germany). Quantification was based on the peak area ratios of the analyte to the internal standard.

References:

S1. Shrestha, R. et al. Exploring the therapeutic potential of mitochondrial uncouplers in cancer. Mol Metab. 2021. 51: 101222.

S2. Starčević, Š., et al., Biochemical and biological evaluation of novel potent coumarin inhibitor of 17β-HSD type 1. Chemico-Biological Interactions, 2011. 191(1): p. 60-65.

S3. Starčević, Š., et al., Synthesis and Biological Evaluation of (6- and 7-Phenyl) Coumarin Derivatives as Selective Nonsteroidal Inhibitors of 17β-Hydroxysteroid Dehydrogenase Type 1. Journal of Medicinal Chemistry, 2011. 54(1): p. 248-261.

S4. Sinreih, M., et al., Chapter Seven - 17β-Hydroxysteroid dehydrogenases types 1 and 2: Enzymatic assays based on radiometric and mass-spectrometric detection, in Methods in Enzymology, T.M. Penning, Editor. 2023, Academic Press. p. 201-234.

S5. Pavlič, R., et al., In the Model Cell Lines of Moderately and Poorly Differentiated Endometrial Carcinoma, Estrogens Can Be Formed via the Sulfatase Pathway. Frontiers in Molecular Biosciences, 2021. 8.

Absorption UV-Vis spectra of compounds **10** and **17b**

**
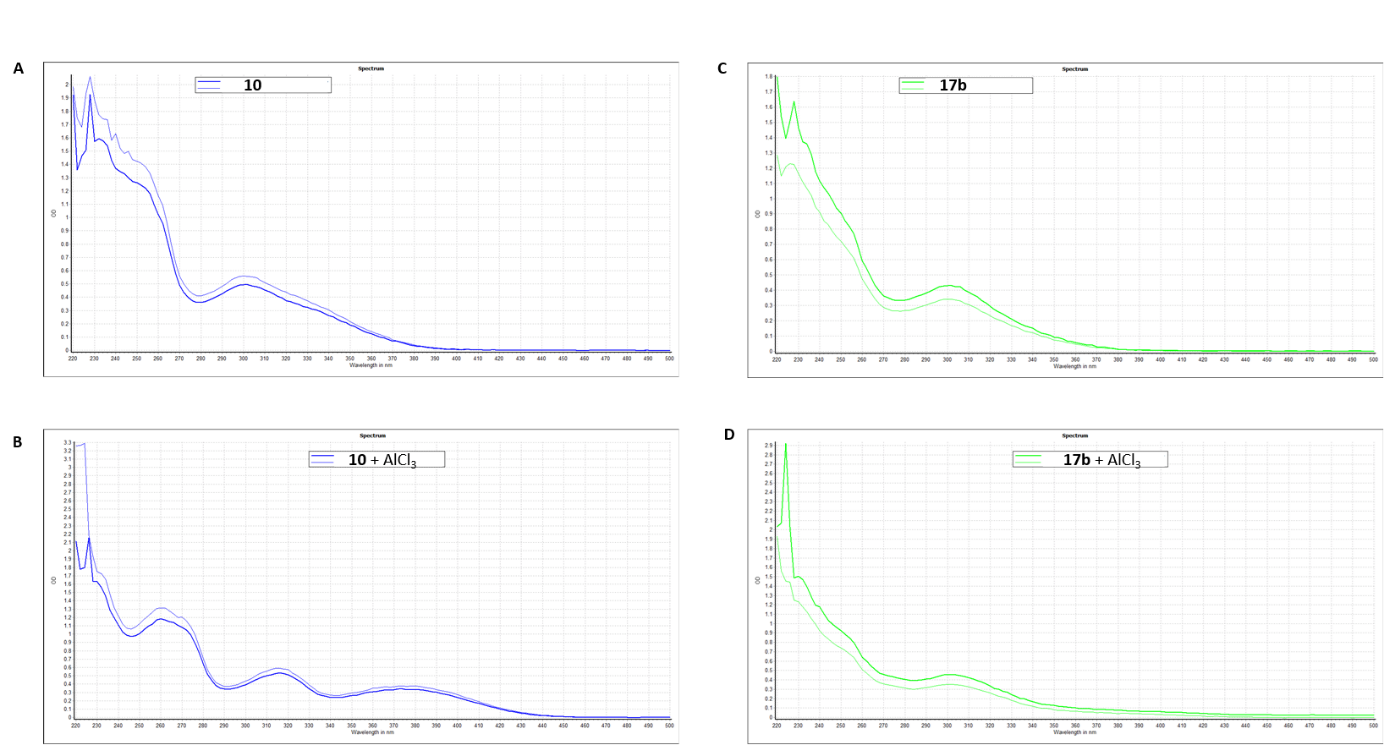
**

SFig. 1. UV-Vis spectra of compound **10** in the absence (**A**) and presence of AlCl_3_ (**B**). UV-Vis spectra of compound **17b** in the absence (**C**) and presence of AlCl_3_ (**D**).

Representative flow cytometry results of the **17c**-induced mitocondrial membrane potential changes.
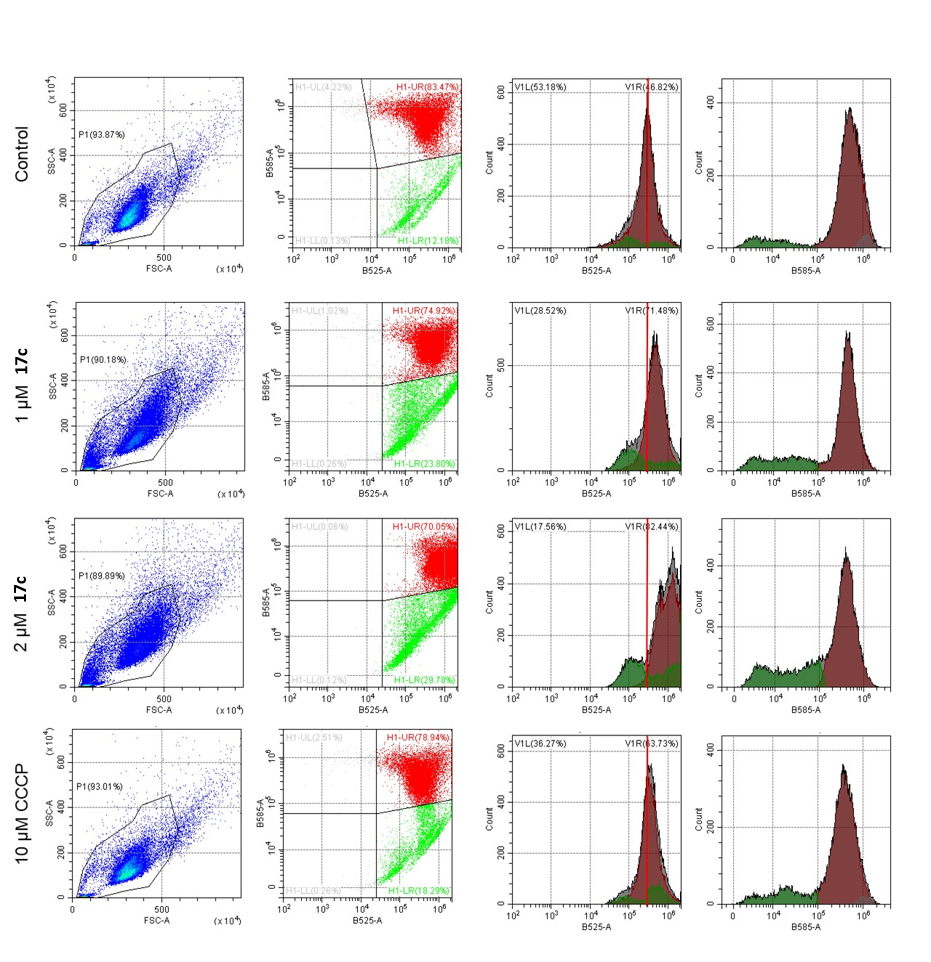


^1^H and ^13^C NMR spectra of the newly synthesized compounds

**13a**


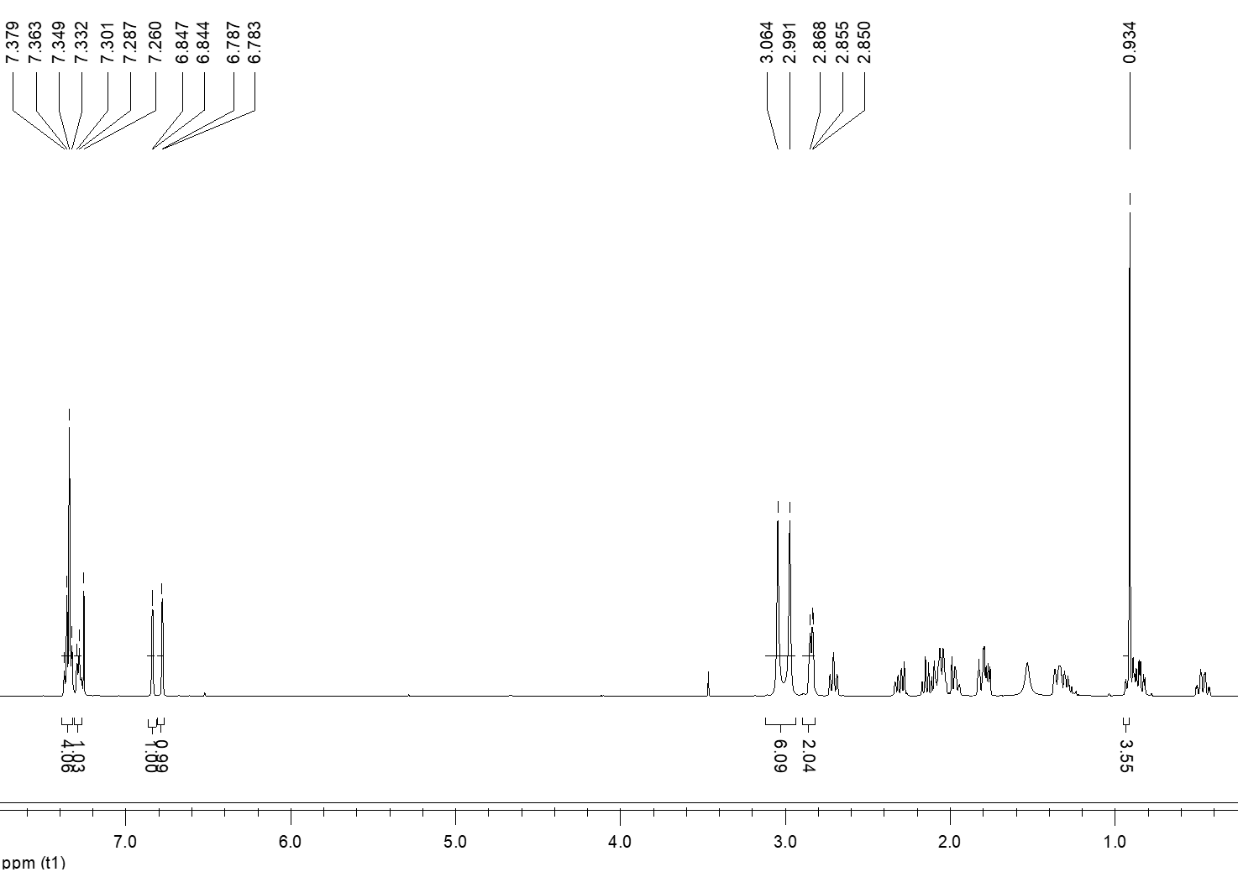


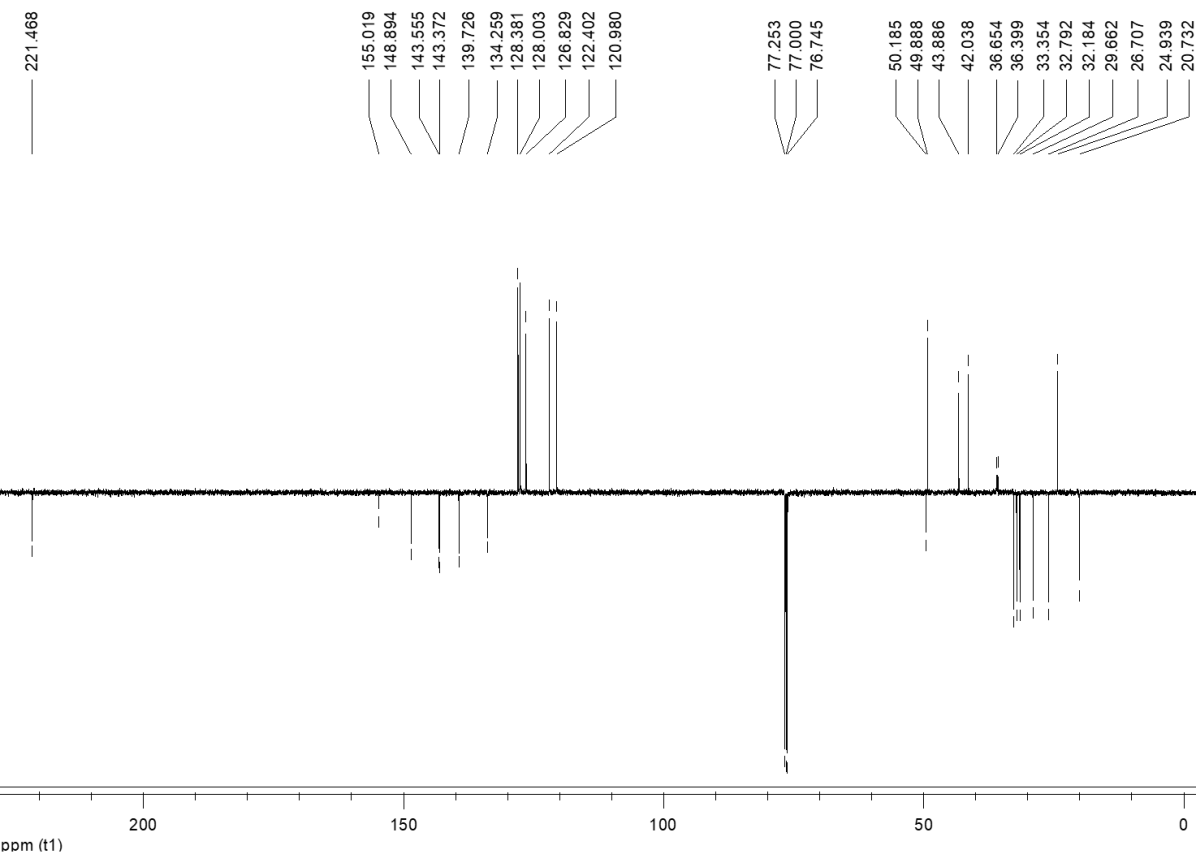

**14a**


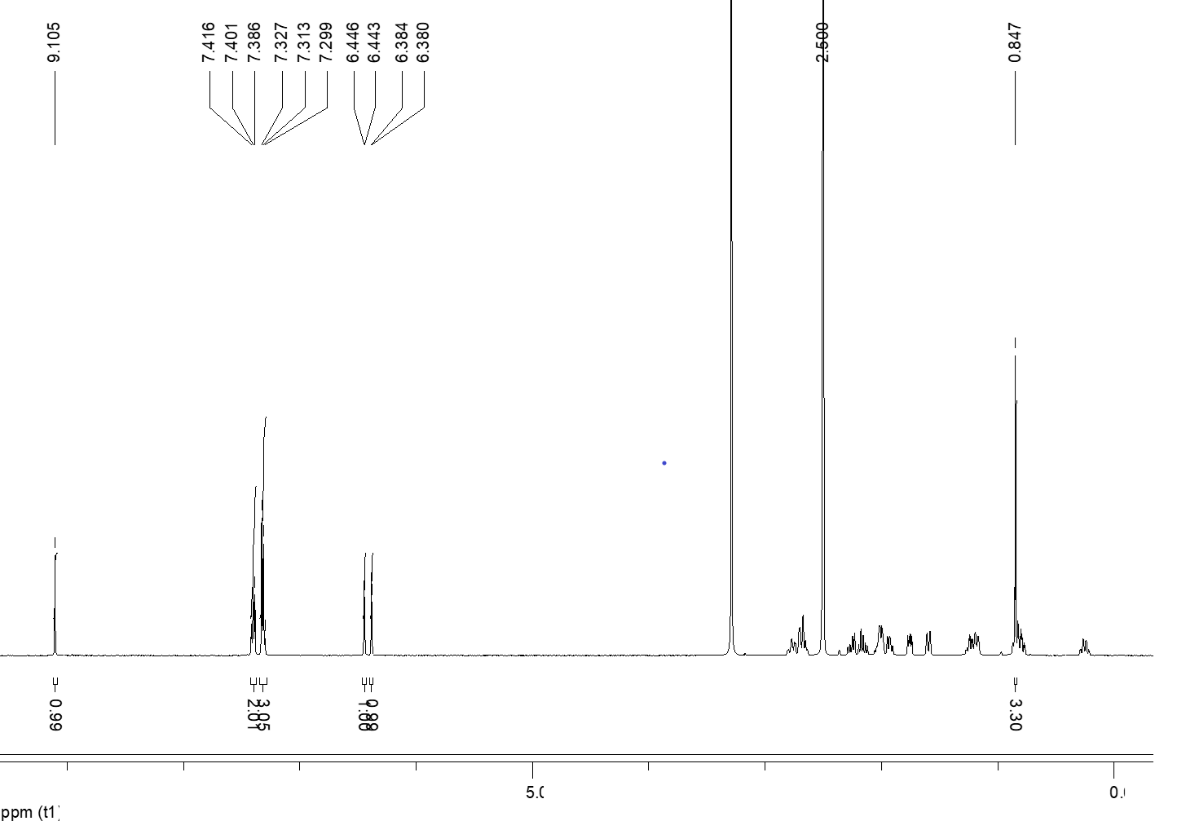


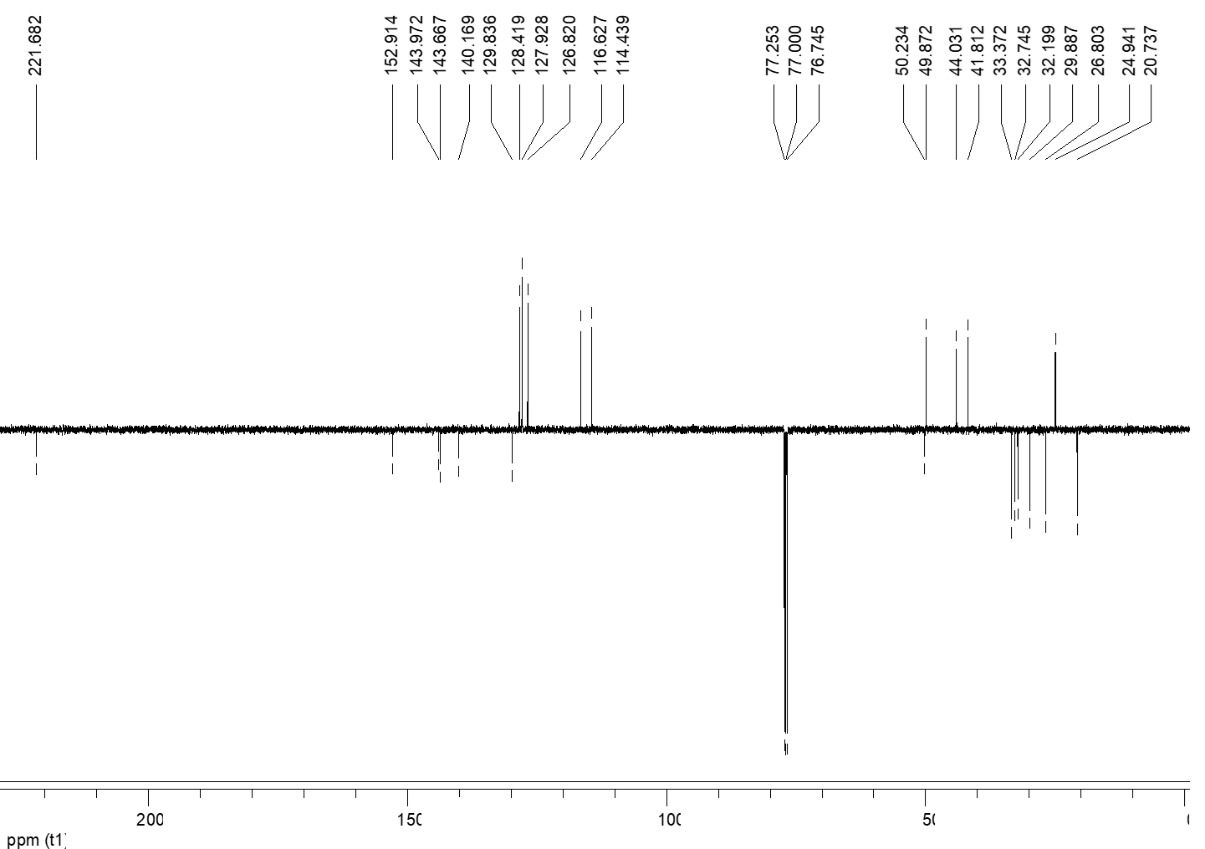

**13b**


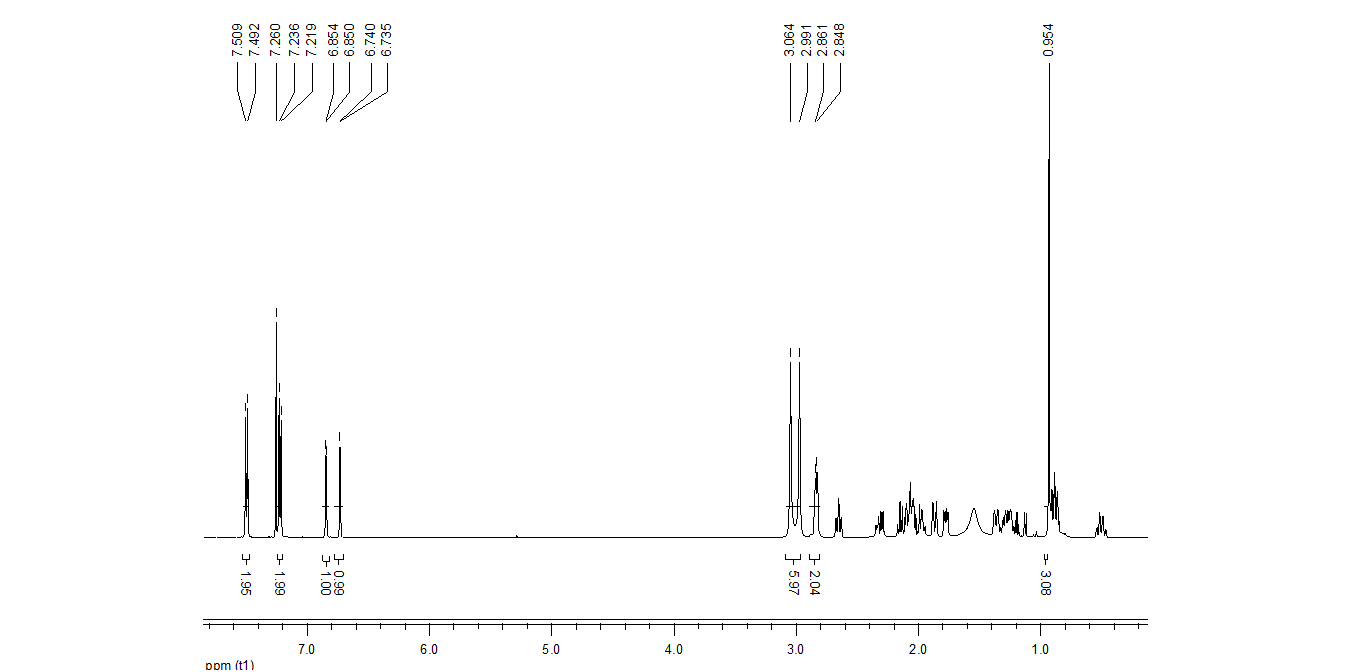


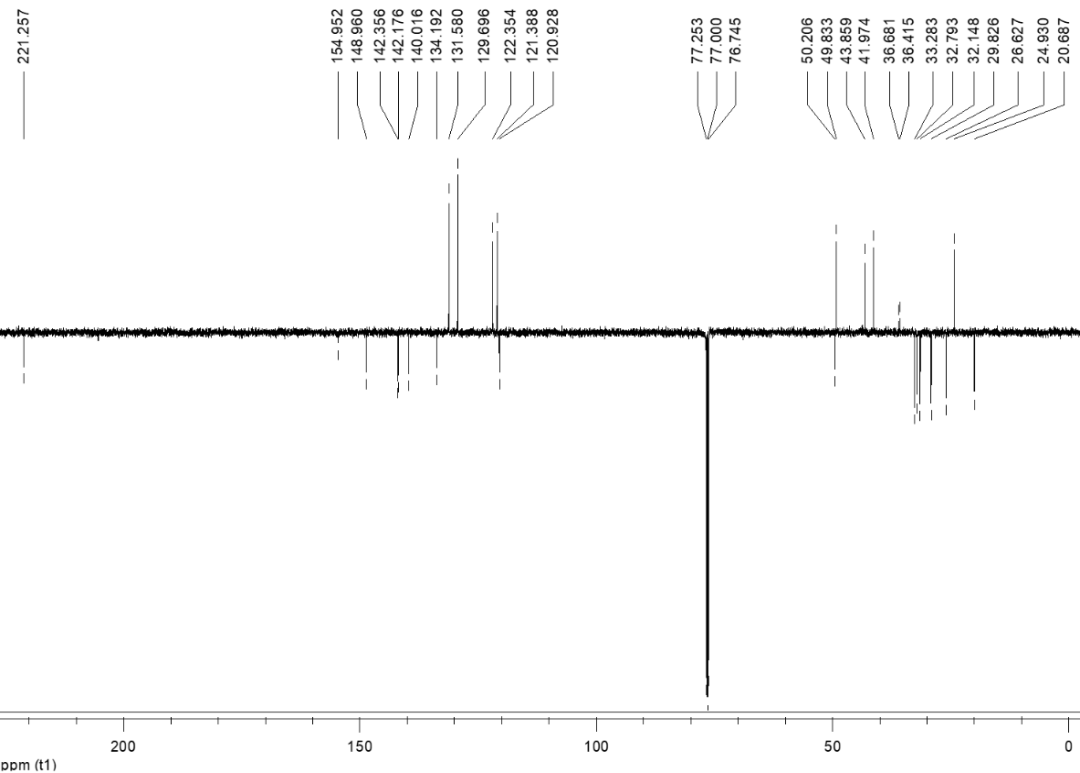

**14b**


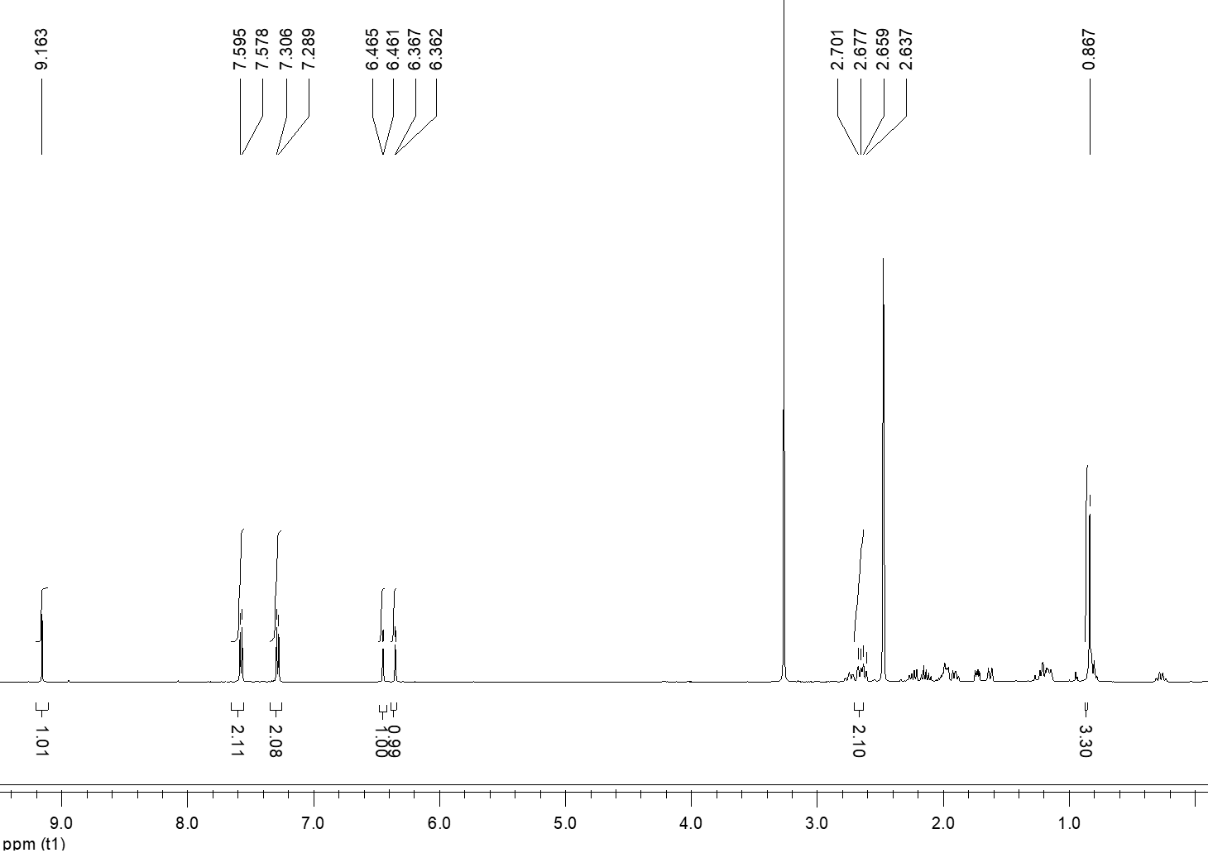


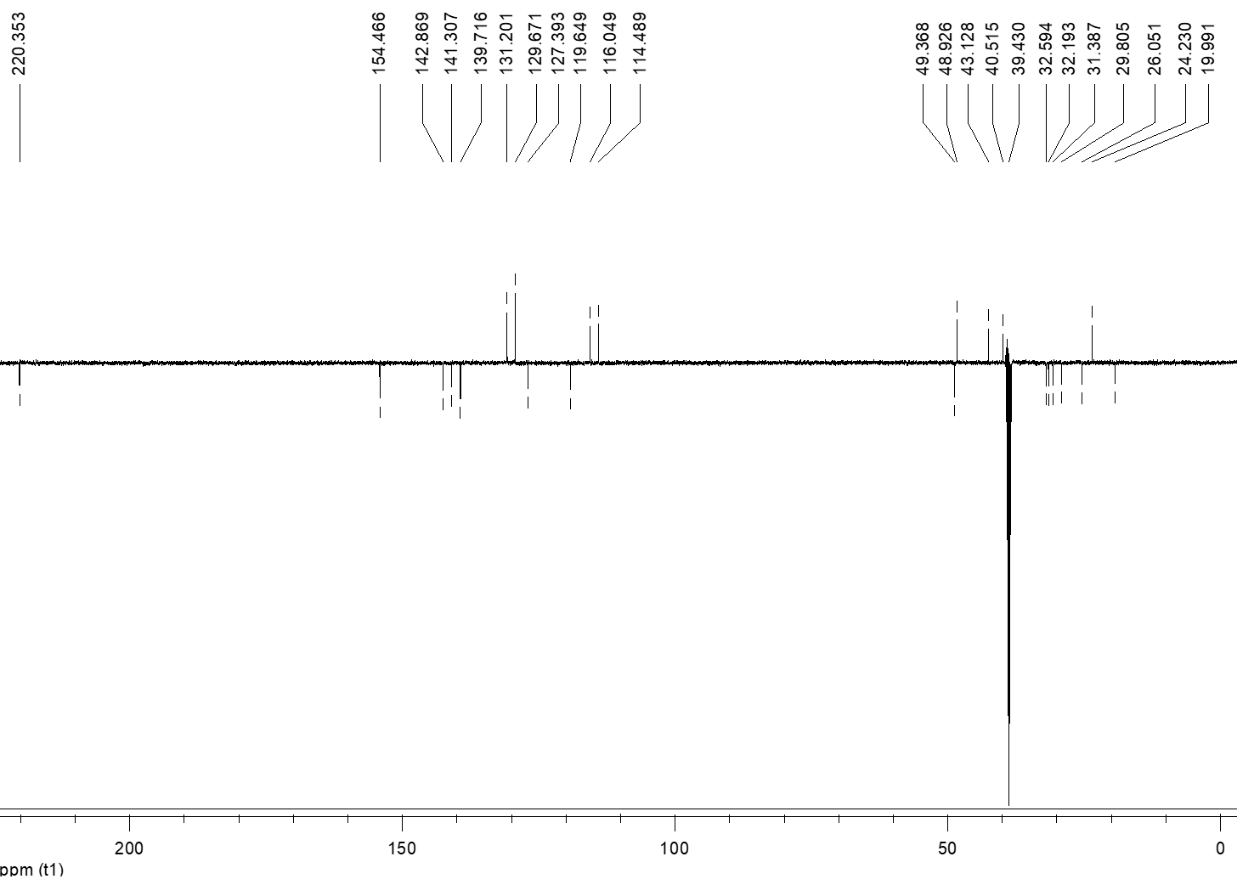

**13c**


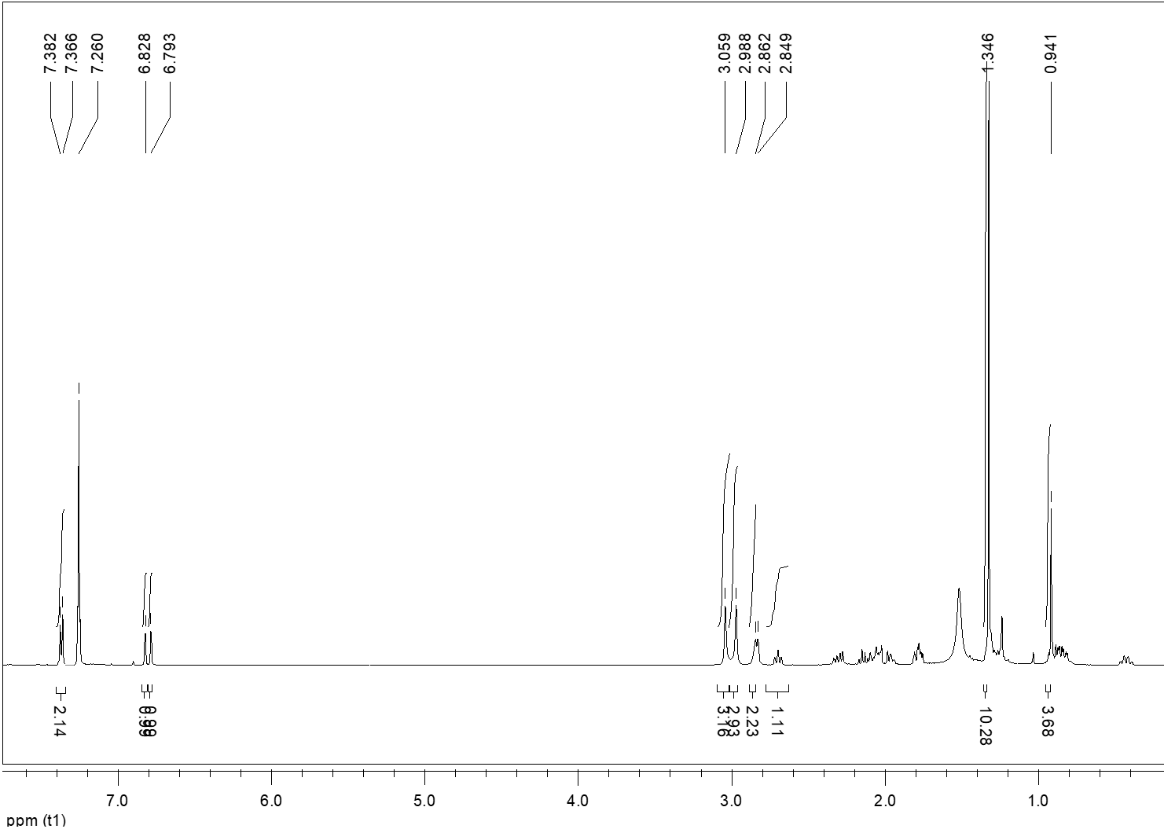


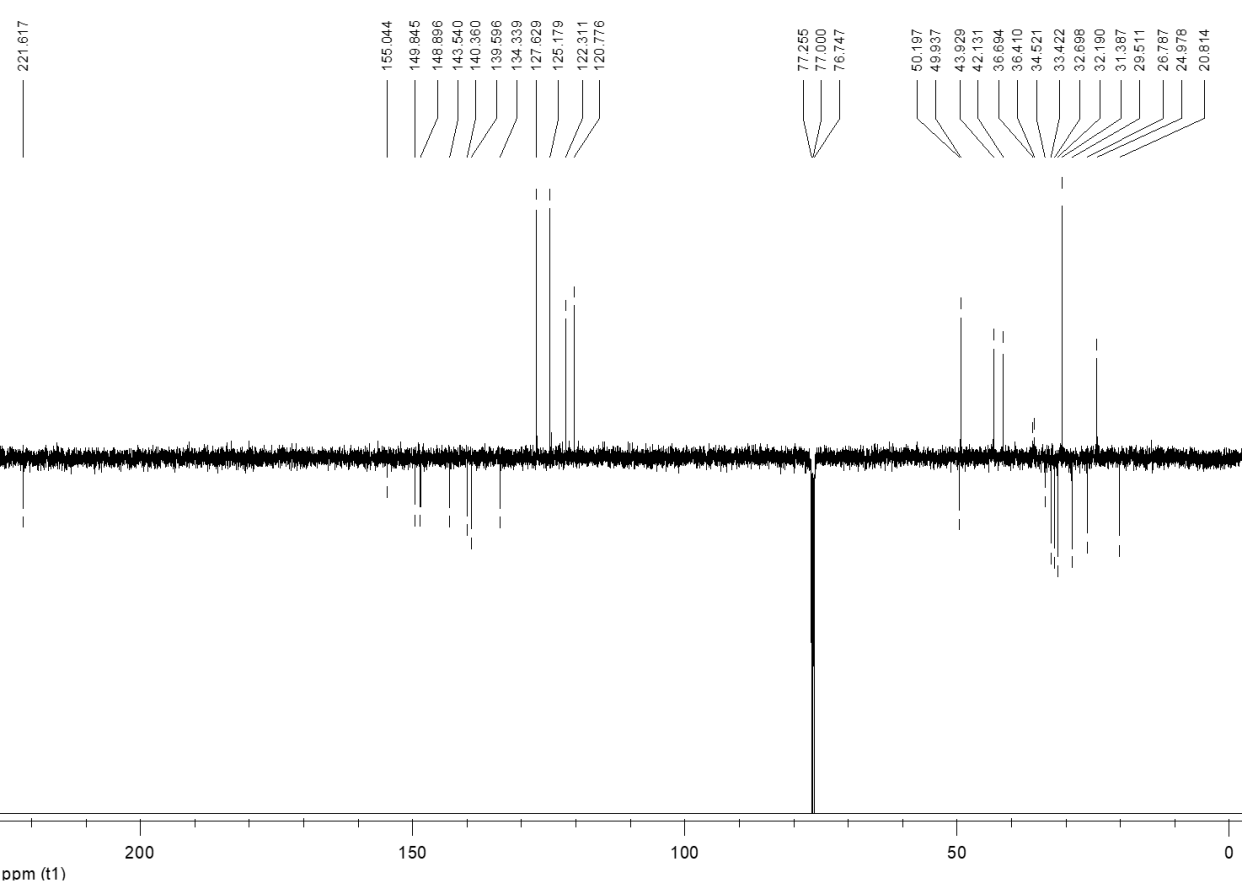

**14c**


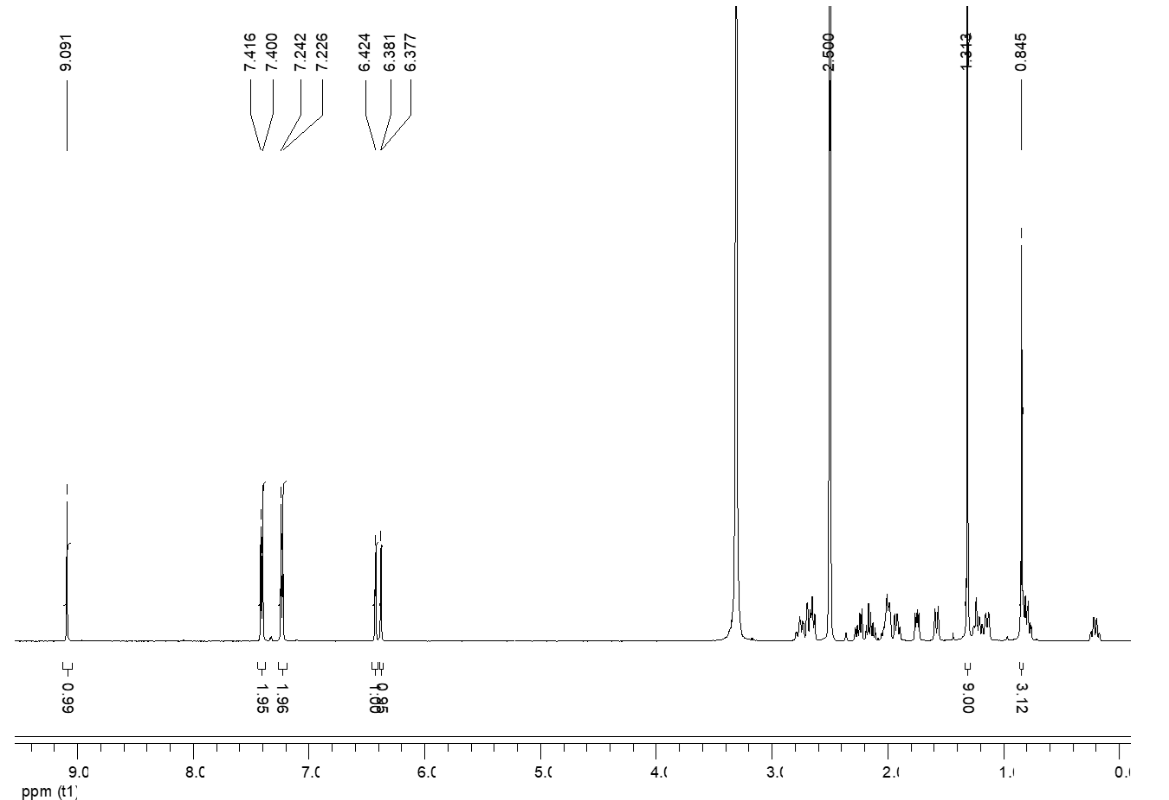


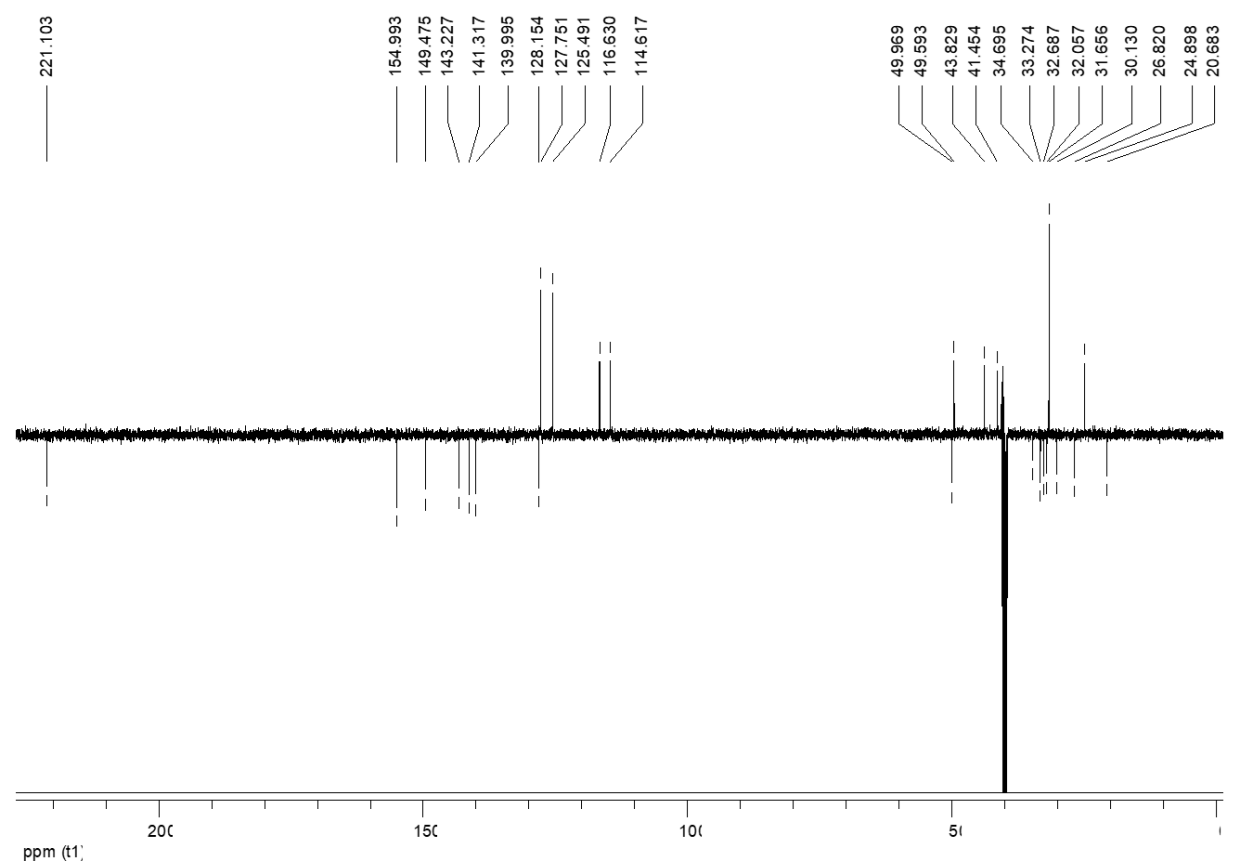

**16**


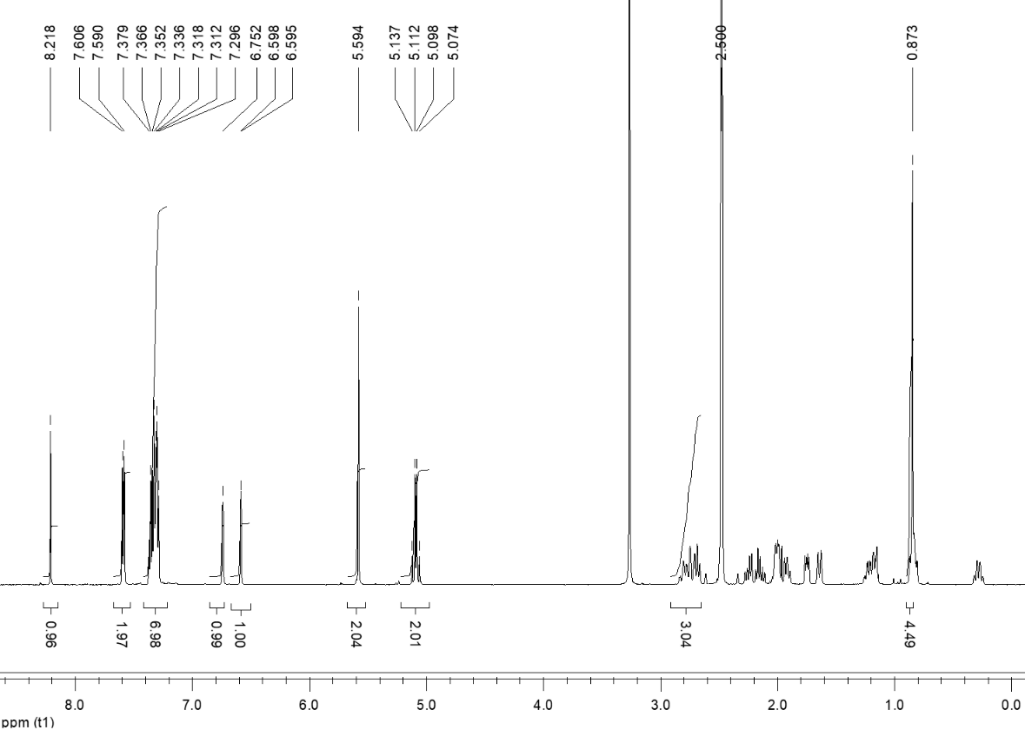


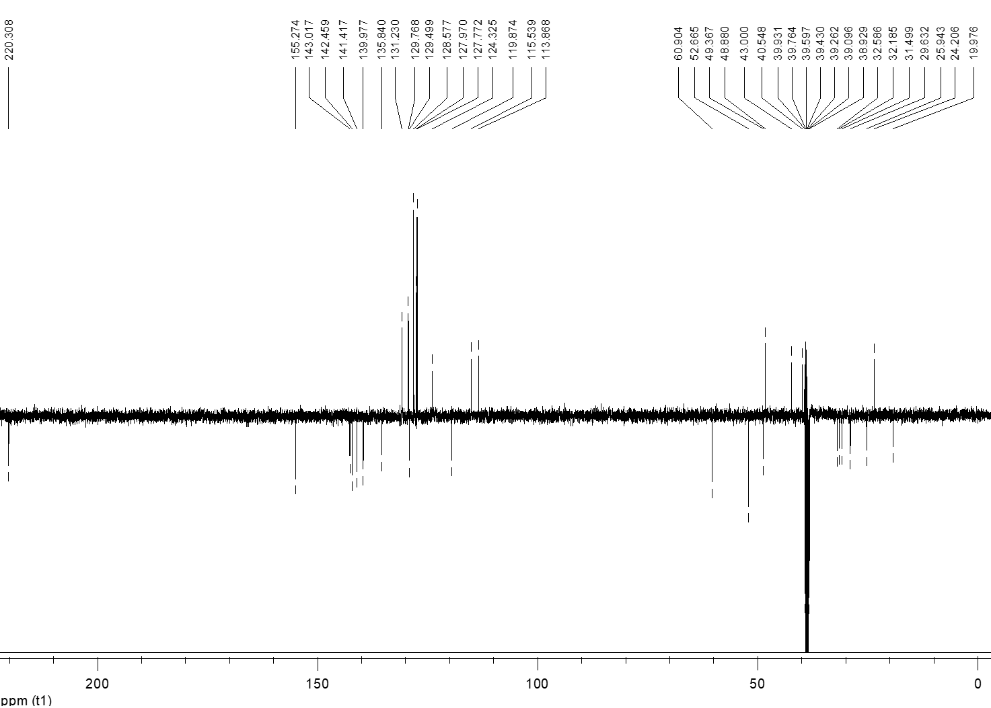

**17a**


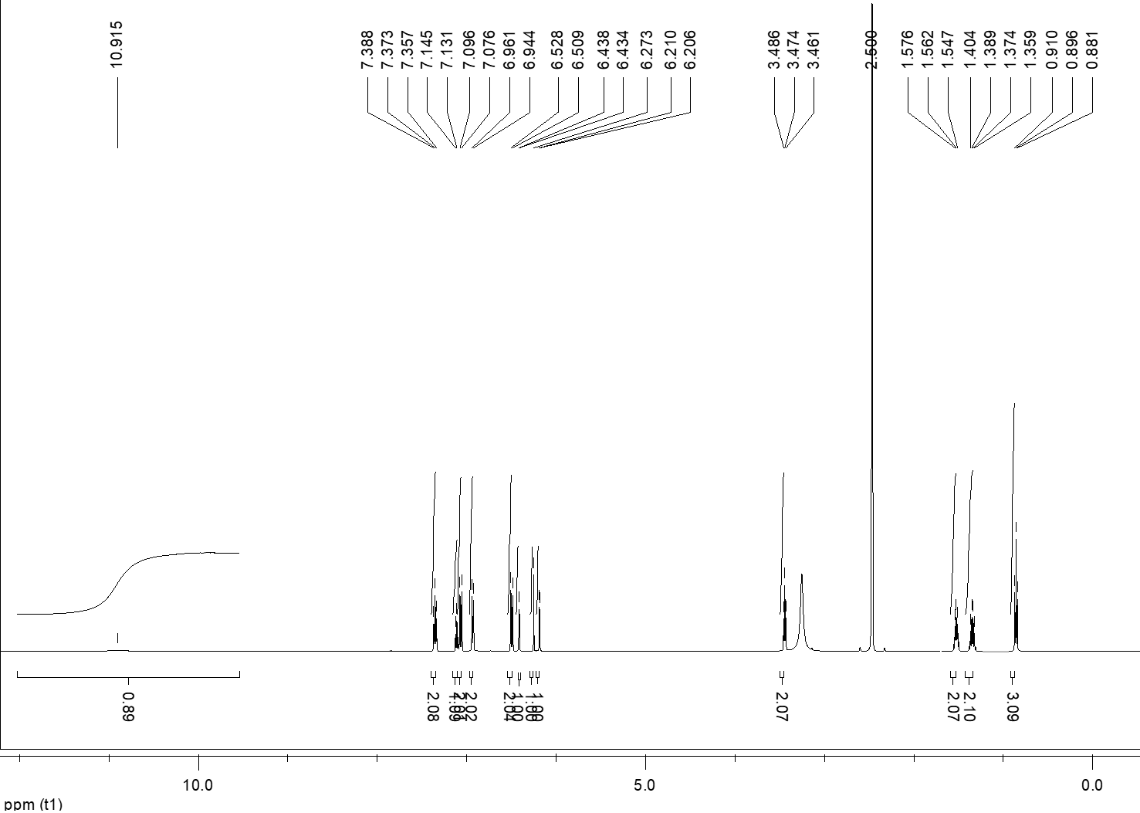


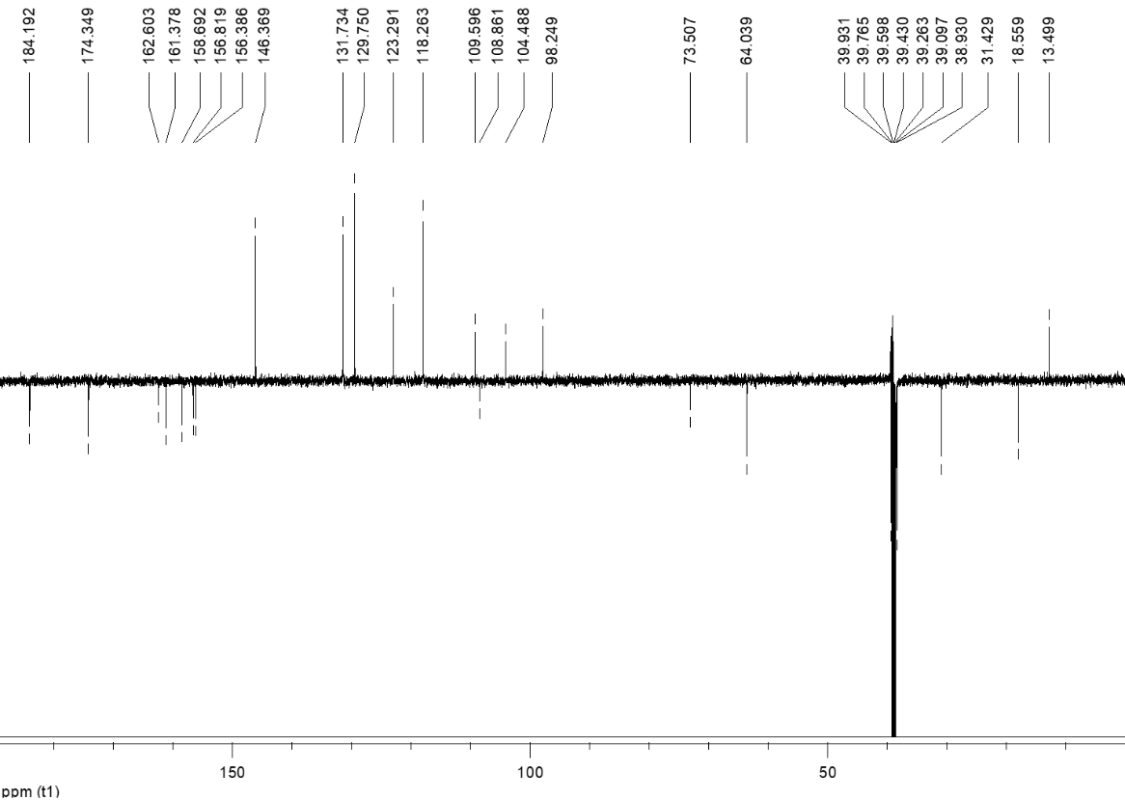

**17b**


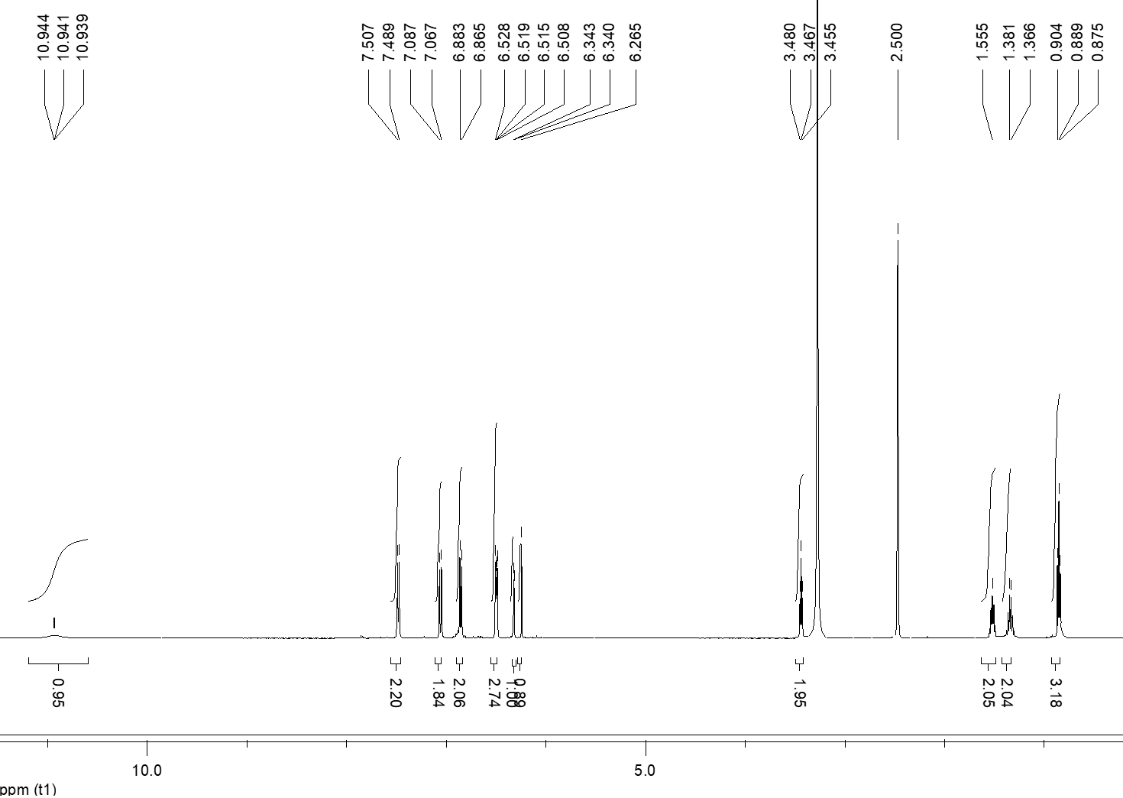


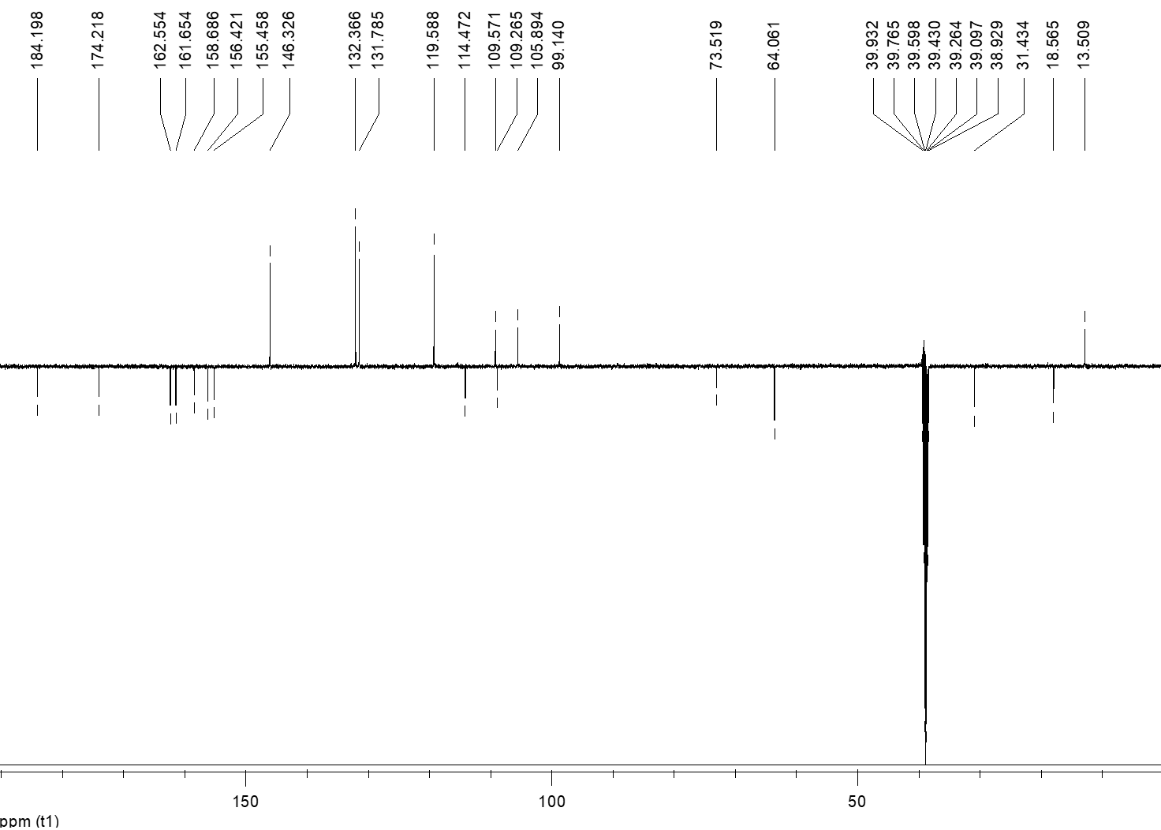

**17c**


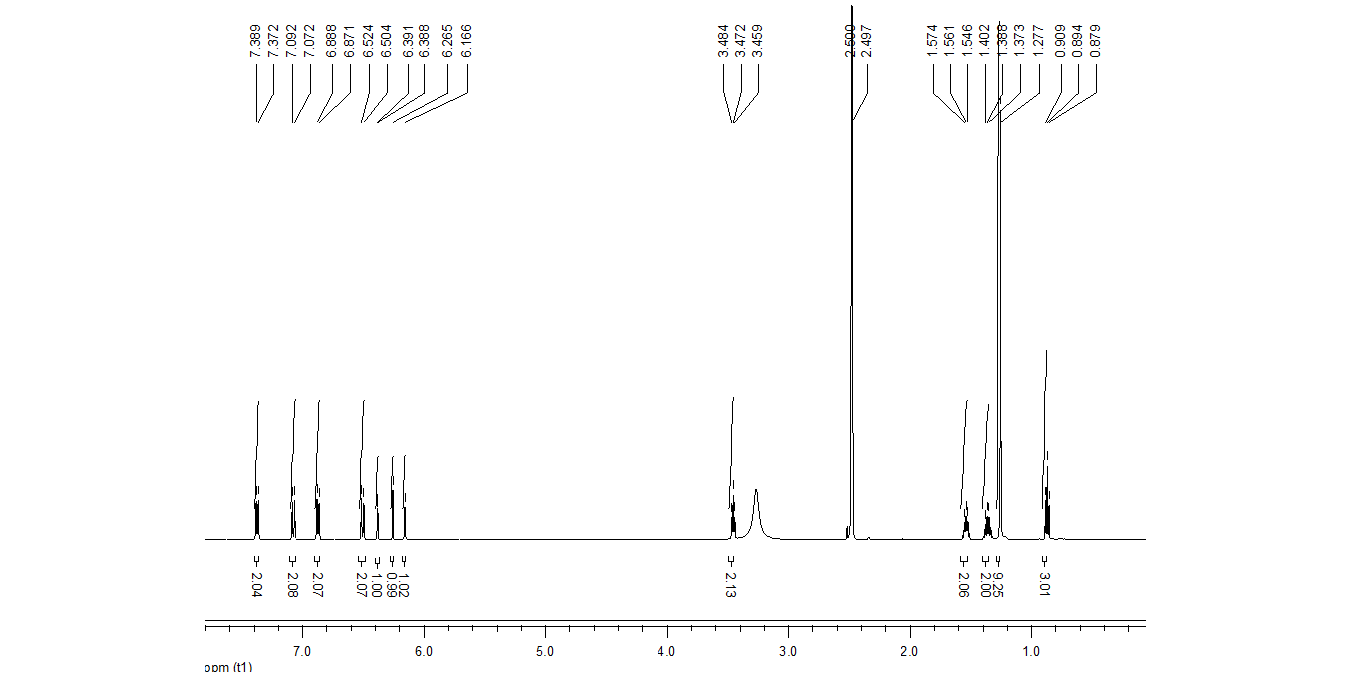


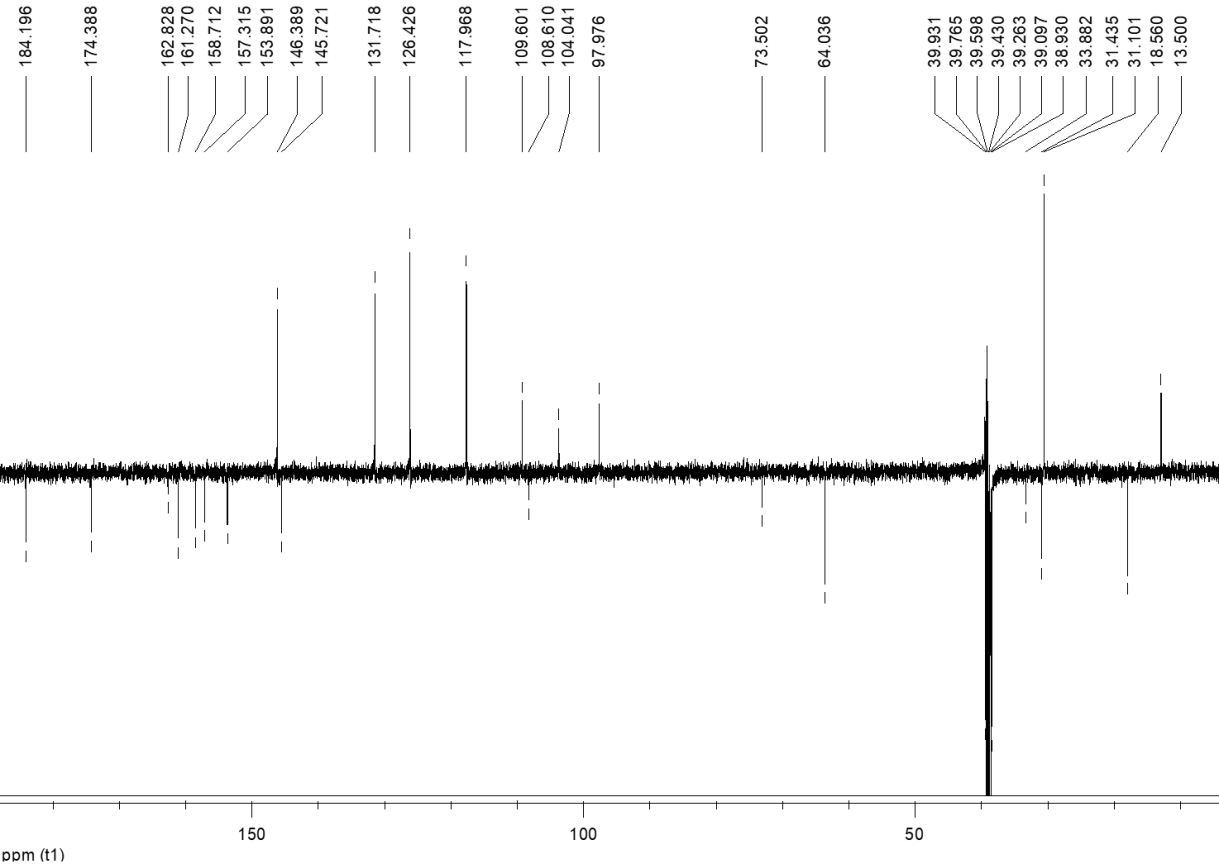


2D NMR spectra of compound **17c**
